# A Biobjective Stochastic Model for Intermodal Supply Chains: Application to the Corn and Soybean Flows

**DOI:** 10.1021/acs.iecr.5c02623

**Published:** 2026-02-19

**Authors:** Marco Marto, Valentina Chkoniya, Eduardo B. Couto, Telmo Pinto, Agostinho Agra, Marco S. Reis

**Affiliations:** † Aveiro Institute of Accounting and Administration and CIDMA Center for Research & Development in Mathematics and Applications, 56062 University of Aveiro 3810-500 Aveiro, Portugal; ‡ Aveiro Institute of Accounting and Administration, University of Aveiro, 3810-500 Aveiro, Portugal; § GOVCOPP Research Unit in Governance, Competitiveness and Public Policies, University of Aveiro, 3810-193 Aveiro, Portugal; ∥ Department of Economics, Management, Industrial Engineering and Tourism, 56062 University of Aveiro 3810-193 Aveiro, Portugal; ⊥ ALGORITMI Research Centre/LASI, University of Minho, Campus de Gualtar, 4710-057 Braga, Portugal; # Departamento de Ciências Matemáticas and Centro de Estudos Matemáticos (CEMS.UL), Faculdade de Ciências, Universidade de Lisboa, 1649-004 Lisbon, Portugal; ¶ Department of Chemical Engineering, University of Coimbra, CERES, 3030-790 Coimbra, Portugal

## Abstract

Recently, several unexpected events of various types (geopolitical, economic, health-related, and weather-related) have had a significant global impact, affecting the lives of millions of people and businesses. Disruptions in supply chains highlight the importance of planning and managing them at various hierarchical levels of decision-making. This paper presents and analyzes supply chain networks (SCNs) for the distribution of soybean and corn in Europe and North Africa, considering the uncertainty of demand and costs along intermodal routes. Transportation and distribution costs for these commodities are minimized by considering the uncertainty of demand and transportation costs. Starting from a deterministic model to establish the SCN, uncertainty is then introduced through stochastic modeling, with the optimization goals redefined accordingly, more specifically in terms of expected cost (EC) and conditional value at risk (CVAR) constraints. The solutions of the bi-objective model, including equivalent average emissions CO_2_, highlight the strategic positions of the port of Itaqui on the supply side and of the port of Sines as distribution hubs (transshipment) in the design of the networks according to the different objectives. The advantageous position of the port of Sines is analyzed in detail, according to the strategic objective of designing a more efficient intermodal SCN for the distribution of soybean and corn.

## Graphical abstract

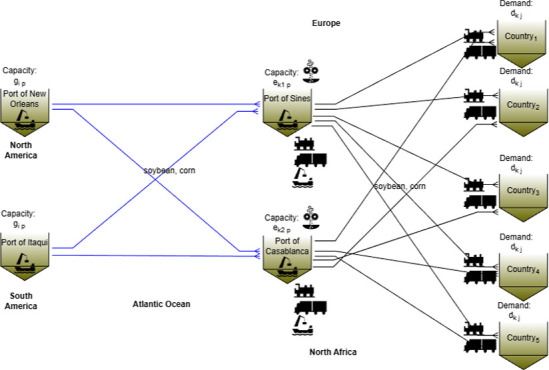

## 1 Introduction

### 1.1 Motivation

This paper focuses on the efficiency of the international supply chain for grains and seeds, with a particular emphasis on food commodities such as soybean and corn. These commodities are primarily distributed to meet demand in Europe, the Middle East, and North Africa (MENA). Soybean and corn are essential commodities in the food chain of Europe and MENA, not only to produce final consumer products but also to feed livestock for human consumption.^1–4^ In the quest to secure access to large-scale plant proteins to sustain meat production, European and North African countries are increasingly dependent on South America’s lower-cost, more productive agricultural lands to supply adequate quantities of these commodities.

In recent decades, soybean has established its dominant role as the primary protein source for meat production in the developed world. With a higher protein content than other seeds, the incorporation of soybean in animal feed results in higher conversion to meat production. The American continents have higher production yields per hectare due to favorable climatic conditions and transgenic seed varieties.^5^ The European Union’s biodiversity regulations severely restrict the use of highly productive transgenic seeds by farmers,^6^ making it difficult for farmers to compete with American agricultural companies. Therefore, the American continents (North and South) have become the primary source of soybean and corn in recent decades.^7^ By analyzing long-term time-series behavior, one can observe that the demand for both commodities is seasonal, with major trends developing over longer time scales. Furthermore, there is a clear correlation between their prices per ton: the price per ton of soybean is more than twice that of corn (see Figure 1).^8,9^

**Figure 1:**
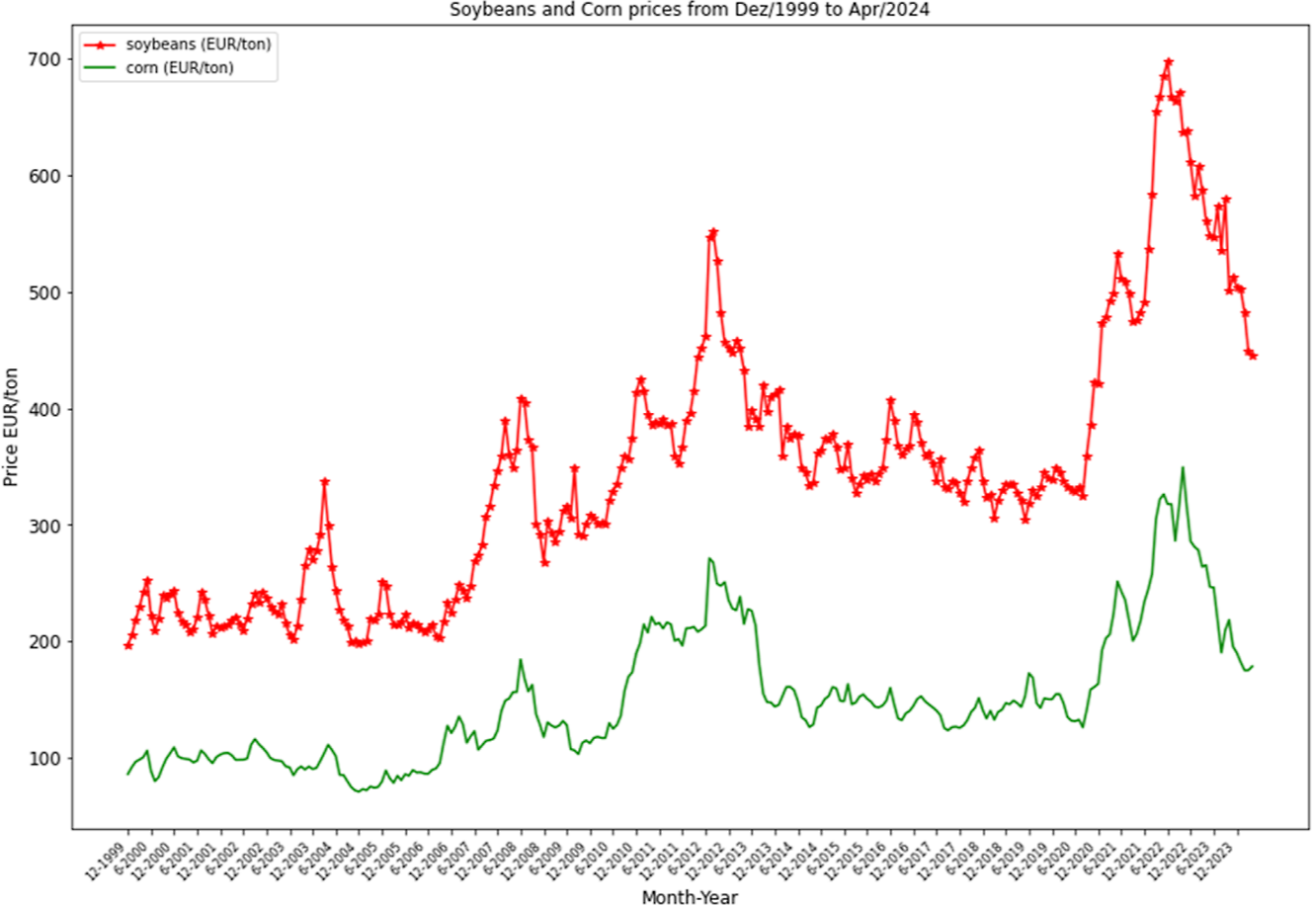
Evolution of prices in EUR per ton of soybean and corn.^8,9^

Since the beginning of the current century, soybean supply chains have shifted toward South American countries (e.g., Brazil, Argentina) as the main suppliers, rather than North American sources (e.g., the U.S.A., Canada).^10^ Corn supply chains are following the same pattern, albeit at a slower pace (see Figures 2 and 3).

**Figure 2:**
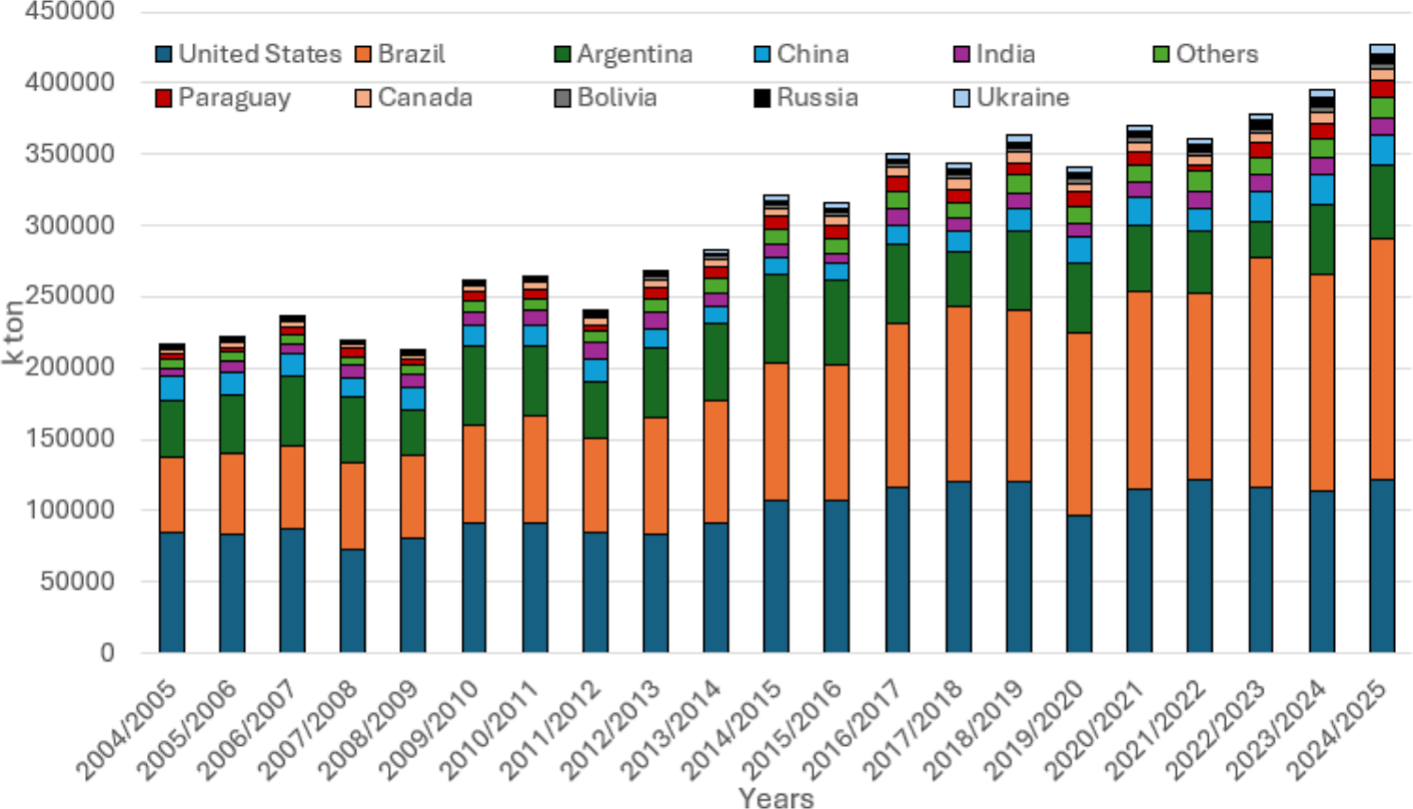
Soybean production from 2004/2005 to 2024/2025 among the top producing countries.^7^

**Figure 3:**
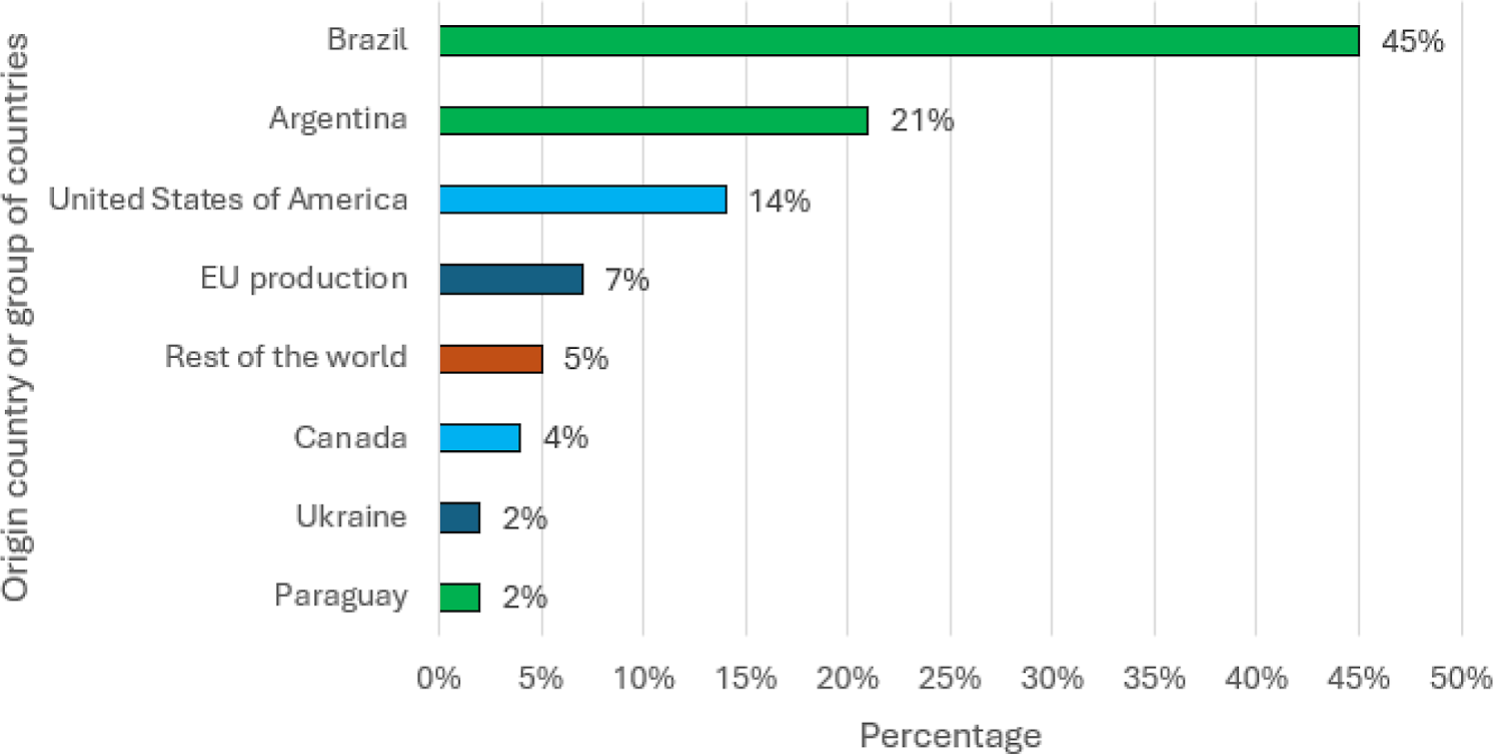
Percentage of soybean, soy meal, and soy oil entering the EU27 in 2021 by origin (adapted from the IDH Soy Monitor report^10^). Bars representing North American countries are depicted in light blue, while those representing South American countries are depicted in green.

The movement of large quantities of grains and seeds between continents relies on an efficient and specialized maritime transport fleet. These are bulk commodities that are transported in specialized vessels known as dry bulk carriers. As demand for these commodities steadily grows, the efficiency of the supply chain becomes critical, with an increasing number and size of vessels required to meet the growing demand.^11^ Bulk carriers come in various sizes, ranging from small coastal ships to massive ocean-going carriers. Large bulk carriers, classified as Panamax size and larger, require deep-water quays. These quays are specifically constructed to accommodate the deep drafts of these ships, ensuring safe and efficient docking, and are typically located in deep-water ports, which have a minimum depth of approximately 12 m.

The ports must also be equipped with advanced logistics infrastructure, including large, fast handling systems and extensive storage facilities, to facilitate cargo loading and unloading. The depth and length of the quays are critical factors, as they determine the size and number of ships that can be accommodated simultaneously.^12^ The modernization of European ports has not always kept pace with the rapid evolution of vessels. While there have been significant efforts to upgrade port infrastructure, the transition can be slow and complex due to various factors, including regulatory requirements, environmental concerns, and the sheer scale of the projects. Still, many European ports managed to adapt to the increasing size of modern vessels, which required significant investments in deeper drafts and more extensive facilities.^12^

Intermodality involves the use of multiple combined modes of transportation (e.g., ocean, rail, waterway, road) to move goods efficiently. For bulk food products, such a combination of transportation modes can reduce costs and improve distribution speed. Shifting from road to rail or waterways can also reduce carbon emissions. Furthermore, ports are improving their rail connections to support intermodal transport, which is vital for the agri-food trading sector.^13^ Food commodities are traded on a large scale worldwide. For each specific trade operation, the supply chain design aims to identify the optimal overall economic and sustainable solution. The design involves several phases, which are briefly referred to next.

The first is the intercontinental phase, which uses large bulk carriers to transport goods across the oceans. These vessels are designed to efficiently transport large quantities of bulk commodities over long distances, typically in the range of 80,000 to 150,000 t. In the second phase of coastal transshipment, goods are often transferred from bulk carriers to smaller, handy-size vessels when the carriers reach a major port. Handy-size ships are better suited for navigating coastal waters and accessing smaller ports that larger vessels cannot reach. Once the goods reach the coastal ports, they are transported inland by rail and road networks.^14^ This third phase ensures that the goods reach their final destinations, such as processing plants or distribution centers.

At each of the three stages referred to above, operations should be optimized to minimize delays, ensure cost-effectiveness, and promote sustainability. Seamless coordination among different modes of transport is essential to maintain the flow of goods, using appropriate handling systems and infrastructure in ports and along transportation routes.

Supply chains change not only for economic reasons but also in response to disruptive political events (on both local and global scales), environmental and social issues, and natural disasters. Therefore, their management needs to be flexible, easily adaptable, and intermodal (e.g., using the sea, roads, and railways) to address any disruptions that may occur.^15^ Intermodality is also important due to concerns about global warming and the reduction of greenhouse gas emissions.^16^ In this context, and in the evolving landscape of the soy and corn SCNs, the port of Sines is considered a leading candidate to supply Mediterranean countries and, eventually, a transshipment hub for the traditional North Sea ports.^17^ Therefore, in this article, the strategic position of the port of Sines was further scrutinized.

### 1.2 Contributions

The scope of this paper is the analysis of the supply chain that delivers the necessary demand for soybean and corn to Europe and North Africa, and the assessment of the role of the port of Sines in this global intermodal SCN. This work introduces an optimization model to explore flexible and intermodal solutions for European and North African destination countries, accounting for the uncertainty in demand and port-related transportation costs. It begins with a deterministic model designed to minimize the costs of transportation and logistics operations within the supply chain. Subsequently, a more realistic stochastic model was developed that incorporated the associated uncertainty in some of the model’s parameters. Both formulations include the intermodality and associated costs, as well as processing and transformations related to perishable commodities (soybean and corn) to preserve their quality and value, primarily at distribution centers. They also account for the shortest path to the destination, considering transportation and port operation costs.

The objective is to minimize the overall supply chain cost, thereby implicitly maximizing the quality and economic value of the goods while minimizing the distance traveled by the goods to their destination. Moreover, intermodal solutions provide alternatives and enable responses to unexpected network disruptions, yielding more robust solutions than existing supply chains.

A CVaR (Conditional Value at Risk; see subsection [Sec sec5.2]) approach was introduced concerning the volumes passing through the port of Sines while minimizing the average SC (supply chain) costs. Some constraints were added to justify the viability and emphasize the port of Sines’ potential role in this SC.

A second objective function was added to assess the model’s environmental friendliness when evaluating the viability of the Port of Sines. The biobjective model aims to minimize average CO_2_-equivalent emissions while limiting the average SC cost to its minimum value increased by a positive percentage.

The strategic advantages of the port of Sines^18,19^ are explored and illustrated in this work with an intermodal supply chain network design (SCND) optimization model. Based on the optimized, feasible solutions for the supply chain network to distribute soybean and corn, decision-makers at different levels can select the best alternative.

## 2 Literature Review

SCND models have already been developed to account for the supply of various commodities. Soybean supply chain management and sustainability were reviewed by Jia et al.^20^ Economic, social, and environmental objectives are often closely linked to sustainability. A conceptual model was proposed, and four main topics were analyzed: land-use policies, value-chain governance, CO_2_ emissions reduction, and barriers (e.g., demand-related). A Benders decomposition approach in an SCND optimization to model wheat distribution in Iran was studied by Naderi et al.^21^ The focus was solely on the distribution of wheat in Iran, with no consideration given to uncertainty. A supply chain network model for perishable products, incorporating location, inventory, and routing decisions, along with a two-phase methodology for integrating sustainability, was developed by Biuki et al.^22^ The authors employed possibilistic programming and fuzzy uncertainty to solve the problem, utilizing a parallel hybridization of genetic algorithms and particle swarm optimization. They concluded that as sustainability requirements increase, transportation volumes decrease; however, they did not use a real-world case study.

In 2009, Mohammadi Bidhandi et al.^23^ proposed a mathematical programming formulation and algorithms for deterministic, multicommodity, and single-period SCND models, including a modified version of Benders decomposition. A multiobjective green SCND model that focuses on the trade-off between total cost and environmental impact for strategic company planning was introduced by Wang et al.^24^ The authors did not account for uncertainty and considered only a single period. Green supply chain models are often associated with environmental concerns, goals, objectives, or targets.

On the other hand, an efficient Lagrangian heuristic algorithm for solving the mixed-integer programming model minimizing the total network costs, such as transport costs, lead time costs, inventory costs, and operating costs, of a two-level, deterministic, one-period, multicommodity SCND problem has been proposed by Sadjady and Davoudpour.^25^ In 2006, Melo et al.^26^ studied the strategic design of SCNDs, considering various aspects, including dynamic horizons, general SCND design, external sourcing, inventory capabilities, product distribution, facility design, available capital, and inventory constraints. Neither of the articles considered uncertainty.

Incorporating uncertainty is crucial for designing effective supply chains, particularly with respect to unpredictable factors such as demand fluctuations and variable costs. In some cases, stochastic programming techniques are employed to model uncertainty.^27,28^ A review paper on SCND, authored by Govindan et al.,^29^ highlights several existing optimization techniques that address robust and stochastic optimization under uncertainty, including recourse-based stochastic programming, risk-averse stochastic programming, robust optimization, and fuzzy mathematical programming.

Analyzing the contributions referred to above, it is evident that the sustainable development of SCNs has been primarily studied in relation to multiple objectives, including economic, social, and environmental factors, as expected. While some studies are multicommodity and multiperiod, the vast majority do not account for uncertainty in critical design parameters, specifically demand and transportation costs. Therefore, in this article, a stochastic approach is developed integrating the conditional value-at-risk (CVaR) to provide a thorough and robust assessment of the economic viability of a new grain terminal for the port of Sines based on the demand of soybean and corn in Europe and North Africa.

Positioning the port of Sines as an operational hub for grain distribution in the European Southwest Atlantic sea coast, particularly aiming at the distribution of multiple large cargoes of soybean and corn destined for European and North African countries. The modeling exercise of this study explores the stochastic approach to sustaining efficiency improvements across all SCN of (South) American origin, which a hypothetical new grain terminal at the port could bring to international food commodities trade, thereby improving food accessibility in markets.

## 3 Data Sources

A data set of historical demand for soybeans and corn in Europe and North Africa from FAOSTAT^30^ was combined with Holt’s additive forecasting model to estimate demand for 2024. These demands were distributed by country (individual demands), using the most recent known proportions. The average of monthly prices was used to estimate the proportional distribution of annual demand over the 12 months of 2024.^8,9^ This multicommodity, multiperiod model does not account for inventory due to the perishability of commodities.

The deterministic problem cost minimization function considers not only transportation costs but also fixed operating costs at the origin port and distribution centers, tariff inflow and outflow costs, and costs associated with port operations (cleaning, drying, and fumigation of commodities).

The maritime transportation costs of the Panamax and Handy-size vessels were adapted from Bernacki (2021),^31^ while experts from the main Portuguese grain transportation companies estimated the terrestrial transportation costs (by road and rail).

The fixed operating costs at the origin ports and distribution centers, as well as the tariff inflow and outflow costs, were estimated using data on some European ports.

Due to the perishability and conservation of the commodities, strict quality control is performed in the distribution centers to ensure that the commodities arrive at the destination with the contracted quality.

Some costs related to cleaning, drying, fumigation, storage, and transformation are considered as average costs for these operations in the main transshipment ports. In the case of soybean, part of the quantities arriving at the transshipment ports are transformed into meal (except for the quantities already transformed arriving from Buenos Aires), considering a loss of product, and a remaining untransformed proportion is also used to satisfy the needs in Europe and North Africa.

The combinations of transportation modes (sea, road, and rail) are determined according to the associated costs and shortest paths using the roads from Google Maps API (routingpy library), the rails from RNE (2025) are introduced,^32^ and the sea routes from the searoute library in Python. Several other useful libraries in Python were also utilized, including networkx and geopandas.

The CO_2_-equivalent emissions, which refer to emissions of CO_2_, CH_4_, and N_2_O were estimated for transportation mode combinations using information from the Climatiq Web site.^33^ For more details about the calculation of the parameters and their sources, see Supporting Information.

## 4 Problem Description

The main aim of this study is to develop a SCN that can meet the demand for soybean and corn in Europe and North Africa while minimizing costs and greenhouse gas emissions. The study also empirically explores, through model-based results, the strategic role of the Port of Sines as a transshipment hub within this SCN, thereby supporting the case for constructing a dedicated grain terminal at this location.

The following candidate supply ports are considered in the design of the SCN to distribute corn and soybean: one in North America (New Orleans) and four in South America (Itaqui, Santos, Montevideo, and Buenos Aires). Supply capacity is estimated based on the required demand in Europe and North Africa.

Due to its perishability, soybean comes from supply ports in grains or already transformed into meal, as is the case of soybean from Buenos Aires, and are transported by vessels (e.g., panamax) to candidate distribution centers with sufficient capacity in their terminals. If it was not transformed into meal before, a percentage of soybean is transformed into meal when the commodities arrive at the distribution centers (transshipment ports). In this process there are some losses, although they are of small magnitude. Both commodities are subject to cleaning, drying, and fumigation in distribution centers to ensure their quality and value.

Afterward, they are transported to their final destinations (in most cases, the geographical centroids of countries’ main shapes are used; otherwise, the capital cities are used). In this final step, the intermodal network is utilized, which can comprise roads, rails, or sea routes (utilizing trucks, trains, or handy-size vessels). Whenever the mode of transport changes, an associated cost is incurred. The objective function considers all the costs related to the transportation demand quantities from the supply port to the destination country and some additional processing costs related to the use of ports and terminals, processing commodities, and use of transport combination modes.

The monthly variation of flows and their prices over the course of a year has been considered. The perishability of the commodities precludes holding inventories for more than a few days (due to loss of quality and value), so it is assumed that there is no inventory. Demand and some port-related transportation costs can be estimated under assumed uncertainty. Uncertainty can be caused by many factors, including environmental, economic, social, and political conditions in ports (e.g., origin ports and logistics management of transshipment ports in terminals) and destinations.^27,28^ To address such uncertainty, the nominal scenario is considered alongside 144 additional scenarios that account for uncertainty in demand and transportation costs.

The decision variables for the transshipment-based problem consist of (for each month): the binary variables that determine which origin ports will supply soybean or corn to Europe and North Africa; the flow variables that determine the flow of soybean or corn from each origin port; the binary variables that determine which distribution centers will receive the grains in Europe or North Africa and distribute them to the destination countries; and the binary variables that determine which distribution centers and transportation mode combinations will deliver the required soybean and corn to the destination countries.

Considering the demand for each European and North African country, the supply capacity at origin ports is assumed to be proportional to historical production in North and South America and the capacity of distribution centers based on countries’ demand. The model constraints ensure that the soybean and corn flows arriving at distribution centers from one or more origin ports are sufficient to satisfy demand in destination countries, using the most efficient combination of transport modes available in the network.

The following list summarizes the model assumptions considered: (i) the necessary European and North African demand for soybean and corn is supplied by ports located in North America (New Orleans) and South America (Itaqui, Santos, Montevideo, Buenos Aires); (ii) the supply capacity for soybean and corn at the ports in North and South America is limited by the historical average proportional distribution of production in North and South America multiplied by the required demand; (iii) the capacity to receive corn and soybean at distribution centers (transshipment ports) is sufficient to supply the demand in destination countries; (iv) soybean is transported from the ports of origin in North America and South America to the ports of transshipment in Europe or North Africa in grain form, except when the port of origin is the port of Buenos Aires, where it is already completely transformed into meal; (v) if soybean arrives unprocessed, a portion is always converted into meal at the distribution center (transshipment port); (vi) corn is not transformed at any point in the supply chain; (vii) the supply chain network from origin to port of transshipment is exclusively by sea (Atlantic Ocean with Panamax vessels); (viii) the supply chain network is intermodal (e.g., {sea, roads}, {sea, rails}, {sea, rails, roads}—sea with Handy-size vessels), and, in most cases, there is more than one way from a distribution center to a destination country; (ix) due to the perishable nature of both commodities, there is no accumulation of inventory between months, and therefore no inventory is carried over from one month to the next; (x) the uncertainty of the transportation network and routes used in the supply chain in this study is reflected in the transportation costs.

## 5 Problem Formulation

Figure 4 illustrates the supply chain network design (SCND). Designing an optimized supply chain is essential for ensuring the demand required by destination countries *j* from origin ports *i*, while obeying origin capacity constraints *g*_ip_ and minimizing costs (*p* is the product index). European and North African ports may operate as transshipment terminals *Z*_kp_ with capacity *e*_kp_ (*k* refers to the transshipment port) to receive maritime flows from the origin ports, where value-added operations such as transformation, fumigation, and drying of perishable goods may also take place. The transshipment port capacities considered in this work are not limited to meeting the required demand; they play the role of a big-M to determine whether to open, or not, the distribution center. Therefore, the demand of each country can be satisfied by only one distribution center. The commodities (corn and soybean) are transported to the destination countries via the shortest available route, using maritime, road, or rail modes, depending on intermodal alternatives. In this supply chain network design, fixed and variable costs associated with transportation and intermodal operations are considered.

**Figure 4:**
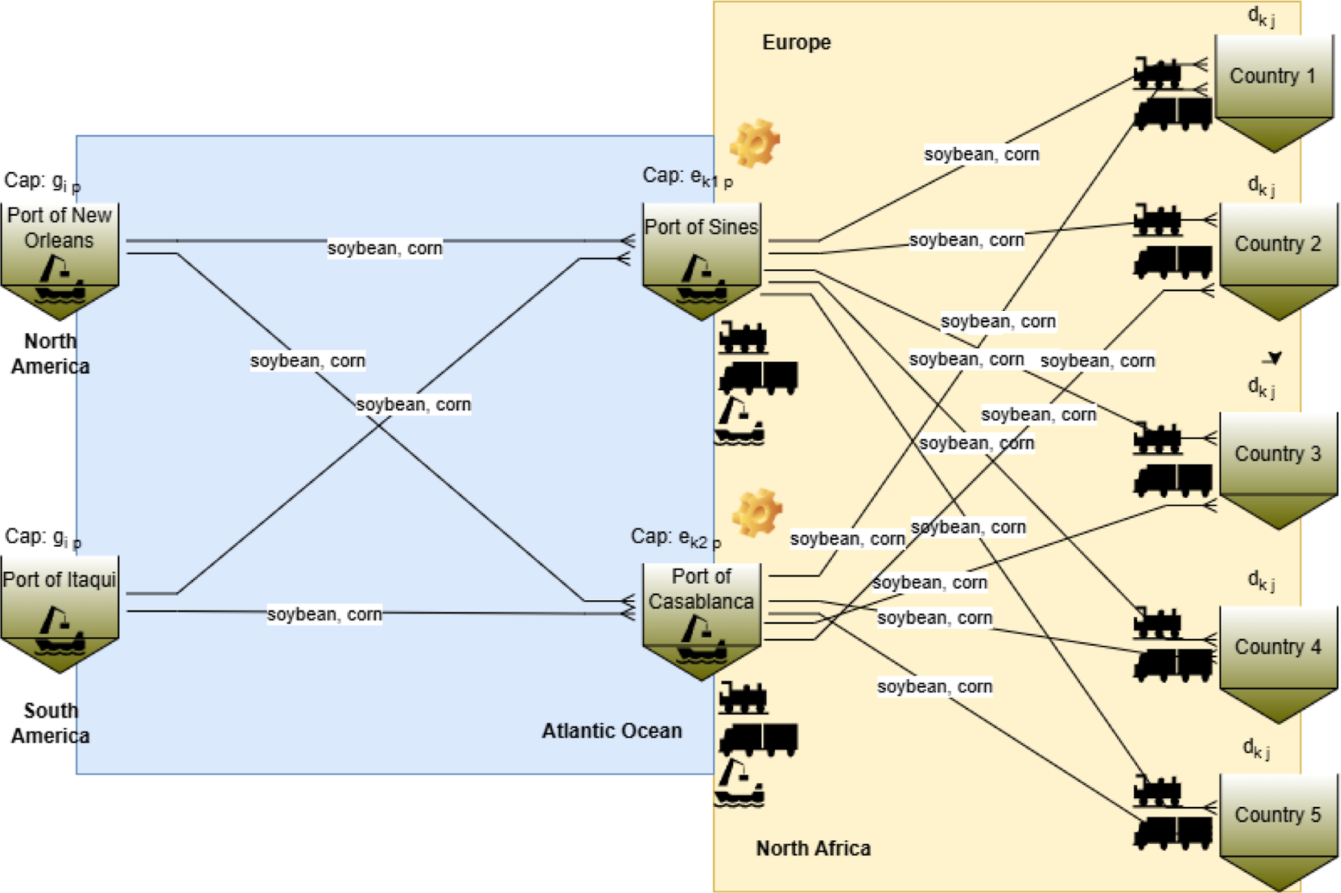
Example of supply chain network design for a given month.

### 5.1 Deterministic Model

This section defines the parameters, their associated indices, the decision variables, the objective function, and the constraints for the deterministic variant of the model. The deterministic mixed-integer programming model is used to solve the transshipment problem in the supply chain network, thereby satisfying demand for soybean and corn in Europe and North Africa.

#### 5.1.1 Parameters and Indices

Indices and general parameters:

- Suppliers (ports of origin) are indexed by *i* ∈ {1, 2, .., *n*};
- Distribution centers (transshipment/intermodal points) are indexed by *k* ∈ {1, 2, ..., *b*};
- Countries of destination are indexed by *j* ∈ {1, 2, ..., *m*};
- Months are indexed by *t* ∈ {1, 2, ..., *ht*};
- Combinations of transportation modes for distribution center *k* and destination *j* are indexed by *l*_*jk*_ ∈ {1, 2, ..., *v*_*jk*_}, where *v*_*jk*_ is the index for the last combination of transportation mode for *j* and *k*;
- Products are indexed by *p* ∈ {1, ..., *np*};
- The demand of the destination country *j* of the product *p* for month *t* is represented by *d*_*jp*_^*t*^ (in kton);
- The proportion of the product *p* from the supplier *i* that is transformed, is represented by ρ_*ip*_.

Fixed costs:

- *fsc*_*i*_: the fixed cost for using a supply port *i* (in EUR);
- *f*_*kp*_: the fixed cost of distribution center *k* for product *p* (in EUR);

Capacities:

- *g*_*ip*_^*t*^: the supplier *i* capacity of product *p* for month *t* (in kton);
- *e*_*kp*_^*t*^: the capacity of the distribution center (transshipment port) *k* for product *p* for month *t* (in kton);

Variable costs:

-
$st_{jkl_{jk}}$ : the variable cost of intermodal transportation combination mode *l*_*jk*_ associated with inflow and outflow operations from distribution center *k* to destination *j* (in EUR per kton of product *p*);
- *c*_*ikp*_: the transportation combination cost between supplier *i* and distribution center *k* for product *p* (in EUR per kton of product *p*);
-
$r_{jkl_{jk}}$ : the unit cost of intermodal transportation combination mode *l*_*jk*_ between distribution center *k* and destination *j* (in EUR per kton of product *p*);
- *fc*_*k*_: the cost for using a distribution center *k* (in EUR per kton of product *p*);

Greenhouse gas emissions:

- *ex*_*ik*_: *CO*_2_-equivalent emissions for transporting product p from origin *i* to the distribution center *k* (in tons of *CO*_2_ per kton of product *p*);
-
$ey_{jkl_{jk}}$ : CO_2_-equivalent emissions by transporting product *p* from distribution center *k* to destination *j* using intermodal transportation combination mode *l*_*jk*_ (in tons of CO_2_ per kton of product *p*);

#### 5.1.2 Decision Variables

The decision variables considered for the deterministic model are the following:

$$\begin{array}{ll} & X_{ikp}^{t}=\{\begin{array}{l} 1,ifsupplier\;i\;is\;serving\;distribution\;center\;k\;\text{for}\;product\;p\;\text{and}\;month\;t\\ 0,otherwise \end{array},\\ & Y_{jkl_{jk}p}^{t}=\{\begin{array}{l} 1,\text{if}\;destination\;j\;\text{and}\;transportation\;mode\;combination\;l_{jk}\\ are\;assigned\;to\;the\;distribution\;center\;k\;\text{for}\;product\;p\;\text{and}\;month\;t\\ 0,otherwise \end{array},\\ & Z_{kp}^{t}=\{\begin{array}{l} 1,\text{if}\;distribution\;center\;k\;is\;operational\;\text{for}\;product\;p\;\text{and}\;month\;t\\ 0,otherwise \end{array},\\ & H_{ikp}^{t}:continuous\;variable\;\text{for}\;the\;flow\;from\;the\;supplier\;i\;to\;the\;distribution\;center\;k,\text{for}\;product\;p\\ & \text{and}\;month\;t\;\left(in\,\;kton\right). \end{array}$$

**Figure 5:**
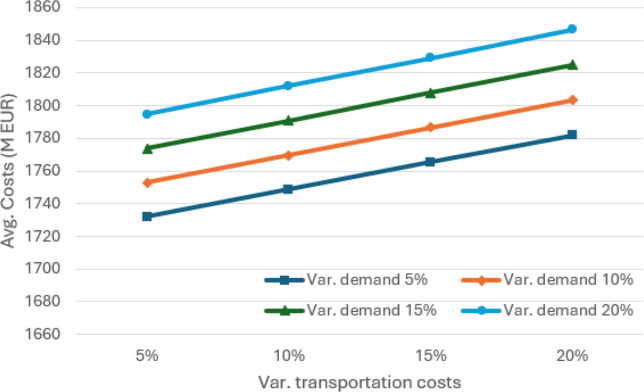
Average cost of the SCND (in millions of euros) for different values of absolute variation in demand and transportation costs, considering θ = 0.95.

**6:**
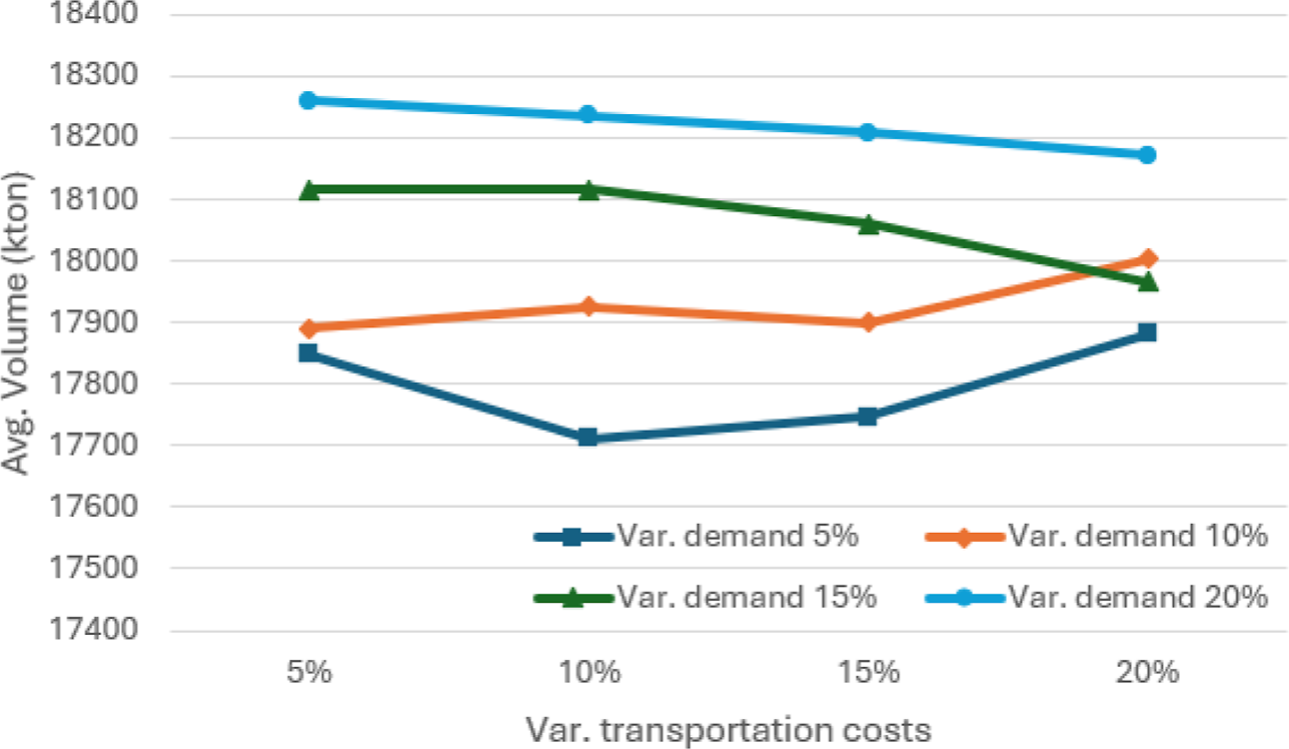
Average volume (in kton) passing through the port of sines for different values of absolute variation in demand and transportation costs, considering θ = 0.95.

**Figure 7:**
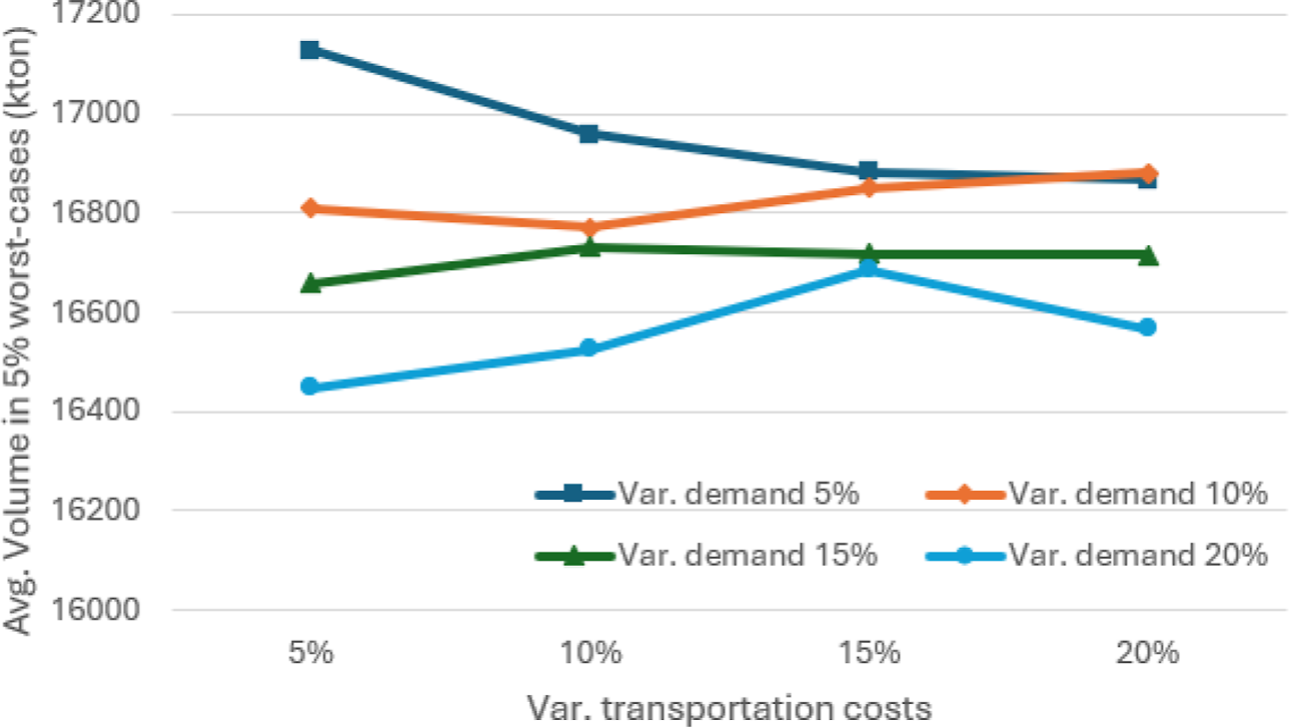
Average volume (in kton) passing through the port of sines in the 5% worst-case scenarios for different values of absolute variation in demand and transportation costs, considering θ = 0.95.

**Table 1:** Stochastic Model Solution that Minimizes the SCN Costs for Corn in Scenario 1 and Month 12^a^

product	origin	distance(km)	distribution center	distance(km)	destination
corn	New Orleans	8896.37	Rotterdam	941.86	Liechtenstein
corn	New Orleans	8896.37	Rotterdam	1443.39	Monaco
corn	New Orleans	8896.37	Rotterdam	110.17	Netherlands
corn	New Orleans	8896.37	Rotterdam	1466.76	San Marino
corn	New Orleans	8896.37	Rotterdam	1414.82	Andorra
corn	New Orleans	8896.37	Rotterdam	2492.12	Gibraltar
corn	New Orleans	8896.37	Rotterdam	2347.80	Iceland
corn	New Orleans	8841.87	Antwerp	2297.56	Estonia
corn	New Orleans	8841.87	Antwerp	2629.90	Russia
corn	New Orleans	8841.87	Antwerp	2359.06	Norway
corn	New Orleans	8841.87	Antwerp	990.83	Denmark
corn	New Orleans	8841.87	Antwerp	1829.91	Lithuania
corn	New Orleans	8841.87	Antwerp	1260.34	Poland
corn	New Orleans	8841.87	Antwerp	2038.86	Latvia
corn	New Orleans	8841.87	Antwerp	3019.44	Finland
corn	New Orleans	8841.87	Antwerp	1956.46	Belarus
corn	New Orleans	8841.87	Antwerp	2244.20	Sweden
corn	New Orleans	8057.41	Lisbon	4135.88	Moldova
corn	New Orleans	8057.41	Lisbon	2172.46	Switzerland
corn	New Orleans	8057.41	Lisbon	2368.86	Italy
corn	New Orleans	8057.41	Lisbon	3543.00	Albania
corn	New Orleans	8057.41	Lisbon	2718.45	Austria
corn	New Orleans	8057.41	Lisbon	2057.39	Belgium
corn	New Orleans	8057.41	Lisbon	2163.92	Luxembourg
corn	New Orleans	8057.41	Lisbon	3718.93	Romania
corn	New Orleans	8057.41	Lisbon	4246.07	Ukraine
corn	New Orleans	8057.41	Lisbon	3773.60	Bulgaria
corn	New Orleans	8057.41	Lisbon	4889.42	Turkey
corn	New Orleans	8057.41	Lisbon	3065.42	Bosnia and Herzegovina
corn	New Orleans	8057.41	Lisbon	3411.01	Montenegro
corn	New Orleans	8057.41	Lisbon	5626.39	Georgia
corn	New Orleans	8057.41	Lisbon	3206.64	Slovakia
corn	New Orleans	8057.41	Lisbon	190.17	Portugal
corn	New Orleans	8057.41	Lisbon	642.69	Spain
corn	New Orleans	8057.41	Lisbon	2870.34	Croatia
corn	New Orleans	8057.41	Lisbon	1551.97	France
corn	New Orleans	8057.41	Lisbon	3136.22	Hungary
corn	New Orleans	8057.41	Lisbon	6077.89	Azerbaijan
corn	New Orleans	8057.41	Lisbon	5867.73	Armenia
corn	New Orleans	8057.41	Lisbon	3628.39	North Macedonia
corn	New Orleans	8057.41	Lisbon	3306.55	Serbia
corn	New Orleans	8057.41	Lisbon	2536.00	Germany
corn	New Orleans	8057.41	Lisbon	2670.50	Slovenia
corn	New Orleans	8057.41	Lisbon	2816.29	Czech Republic
corn	New Orleans	8449.66	Gijon	3844.32	Greece
corn	New Orleans	8528.55	Le Havre	784.32	United Kingdom
corn	New Orleans	8528.55	Le Havre	1019.80	Ireland
corn	Itaqui	5718.43	Lisbon	4135.88	Moldova
corn	Itaqui	5718.43	Lisbon	2172.46	Switzerland
corn	Itaqui	5718.43	Lisbon	2368.86	Italy
corn	Itaqui	5718.43	Lisbon	3543.00	Albania
corn	Itaqui	5718.43	Lisbon	2718.45	Austria
corn	Itaqui	5718.43	Lisbon	2057.39	Belgium
corn	Itaqui	5718.43	Lisbon	2163.92	Luxembourg
corn	Itaqui	5718.43	Lisbon	3718.93	Romania
corn	Itaqui	5718.43	Lisbon	4246.07	Ukraine
corn	Itaqui	5718.43	Lisbon	3773.60	Bulgaria
corn	Itaqui	5718.43	Lisbon	4889.42	Turkey
corn	Itaqui	5718.43	Lisbon	3065.42	Bosnia and Herzegovina
corn	Itaqui	5718.43	Lisbon	3411.01	Montenegro
corn	Itaqui	5718.43	Lisbon	5626.39	Georgia
corn	Itaqui	5718.43	Lisbon	3206.64	Slovakia
corn	Itaqui	5718.43	Lisbon	190.17	Portugal
corn	Itaqui	5718.43	Lisbon	642.69	Spain
corn	Itaqui	5718.43	Lisbon	2870.34	Croatia
corn	Itaqui	5718.43	Lisbon	1551.97	France
corn	Itaqui	5718.43	Lisbon	3136.22	Hungary
corn	Itaqui	5718.43	Lisbon	6077.89	Azerbaijan
corn	Itaqui	5718.43	Lisbon	5867.73	Armenia
corn	Itaqui	5718.43	Lisbon	3628.39	North Macedonia
corn	Itaqui	5718.43	Lisbon	3306.55	Serbia
corn	Itaqui	5718.43	Lisbon	2536.00	Germany
corn	Itaqui	5718.43	Lisbon	2670.50	Slovenia
corn	Itaqui	5718.43	Lisbon	2816.29	Czech Republic
corn	Itaqui	6755.82	Tarragona	3018.03	Egypt
corn	Itaqui	6755.82	Tarragona	980.07	Tunisia
corn	Itaqui	6755.82	Tarragona	545.49	Algeria
corn	Itaqui	6755.82	Tarragona	3066.99	Cyprus
corn	Itaqui	6755.82	Tarragona	1330.87	Malta
corn	Santos	7967.53	Casablanca	393.78	Morocco
corn	Santos	7967.53	Casablanca	2431.74	Libya

a The table shows the distance, in kilometers, between the ports and the capital city or centroid of the destination country. Please note that differences may occur mainly in terms of flows, but also in terms of origin ports and distribution centers, between different months.

**Table 2:** Stochastic Model Solution Minimizes SCN Costs for Soybean in Scenario 1 and Month 12^a^

product	origin	distance(km)	distribution center	distance(km)	destination
soybean	New Orleans	8896.37	Rotterdam	110.17	Netherlands
soybean	New Orleans	8896.37	Rotterdam	2347.80	Iceland
soybean	New Orleans	8841.87	Antwerp	2297.56	Estonia
soybean	New Orleans	8841.87	Antwerp	2629.90	Russia
soybean	New Orleans	8841.87	Antwerp	2359.06	Norway
soybean	New Orleans	8841.87	Antwerp	990.83	Denmark
soybean	New Orleans	8841.87	Antwerp	1829.91	Lithuania
soybean	New Orleans	8841.87	Antwerp	1260.34	Poland
soybean	New Orleans	8841.87	Antwerp	2038.86	Latvia
soybean	New Orleans	8841.87	Antwerp	3019.44	Finland
soybean	New Orleans	8841.87	Antwerp	1956.46	Belarus
soybean	New Orleans	8841.87	Antwerp	2508.46	Greece
soybean	New Orleans	8841.87	Antwerp	2244.20	Sweden
soybean	New Orleans	8090.08	Sines	2316.39	Liechtenstein
soybean	New Orleans	8090.08	Sines	4195.72	Moldova
soybean	New Orleans	8090.08	Sines	1916.62	Monaco
soybean	New Orleans	8090.08	Sines	2232.30	Switzerland
soybean	New Orleans	8090.08	Sines	2428.69	Italy
soybean	New Orleans	8090.08	Sines	3602.83	Albania
soybean	New Orleans	8090.08	Sines	2778.28	Austria
soybean	New Orleans	8090.08	Sines	2117.23	Belgium
soybean	New Orleans	8090.08	Sines	2223.75	Luxembourg
soybean	New Orleans	8090.08	Sines	3778.77	Romania
soybean	New Orleans	8090.08	Sines	2496.31	San Marino
soybean	New Orleans	8090.08	Sines	4305.90	Ukraine
soybean	New Orleans	8090.08	Sines	3833.43	Bulgaria
soybean	New Orleans	8090.08	Sines	4949.26	Turkey
soybean	New Orleans	8090.08	Sines	1314.66	Andorra
soybean	New Orleans	8090.08	Sines	3125.25	Bosnia and Herzegovina
soybean	New Orleans	8090.08	Sines	3470.85	Montenegro
soybean	New Orleans	8090.08	Sines	5686.23	Georgia
soybean	New Orleans	8090.08	Sines	3266.47	Slovakia
soybean	New Orleans	8090.08	Sines	280.90	Portugal
soybean	New Orleans	8090.08	Sines	702.52	Spain
soybean	New Orleans	8090.08	Sines	2930.18	Croatia
soybean	New Orleans	8090.08	Sines	1611.80	France
soybean	New Orleans	8090.08	Sines	3196.05	Hungary
soybean	New Orleans	8090.08	Sines	6137.72	Azerbaijan
soybean	New Orleans	8090.08	Sines	5927.57	Armenia
soybean	New Orleans	8090.08	Sines	3688.22	North Macedonia
soybean	New Orleans	8090.08	Sines	826.24	Gibraltar
soybean	New Orleans	8090.08	Sines	3366.39	Serbia
soybean	New Orleans	8090.08	Sines	2595.84	Germany
soybean	New Orleans	8090.08	Sines	2730.34	Slovenia
soybean	New Orleans	8090.08	Sines	2876.12	Czech Republic
soybean	New Orleans	8090.08	Sines	908.63	Morocco
soybean	New Orleans	8090.08	Sines	2553.62	Libya
soybean	New Orleans	8528.55	Le Havre	784.32	United Kingdom
soybean	New Orleans	8528.55	Le Havre	1019.80	Ireland
soybean	Itaqui	5688.83	Sines	2316.39	Liechtenstein
soybean	Itaqui	5688.83	Sines	4195.72	Moldova
soybean	Itaqui	5688.83	Sines	1916.62	Monaco
soybean	Itaqui	5688.83	Sines	2232.30	Switzerland
soybean	Itaqui	5688.83	Sines	2428.69	Italy
soybean	Itaqui	5688.83	Sines	3602.83	Albania
soybean	Itaqui	5688.83	Sines	2778.28	Austria
soybean	Itaqui	5688.83	Sines	2117.23	Belgium
soybean	Itaqui	5688.83	Sines	2223.75	Luxembourg
soybean	Itaqui	5688.83	Sines	3778.77	Romania
soybean	Itaqui	5688.83	Sines	2496.31	San Marino
soybean	Itaqui	5688.83	Sines	4305.90	Ukraine
soybean	Itaqui	5688.83	Sines	3833.43	Bulgaria
soybean	Itaqui	5688.83	Sines	4949.26	Turkey
soybean	Itaqui	5688.83	Sines	1314.66	Andorra
soybean	Itaqui	5688.83	Sines	3125.25	Bosnia and Herzegovina
soybean	Itaqui	5688.83	Sines	3470.85	Montenegro
soybean	Itaqui	5688.83	Sines	5686.23	Georgia
soybean	Itaqui	5688.83	Sines	3266.47	Slovakia
soybean	Itaqui	5688.83	Sines	280.90	Portugal
soybean	Itaqui	5688.83	Sines	702.52	Spain
soybean	Itaqui	5688.83	Sines	2930.18	Croatia
soybean	Itaqui	5688.83	Sines	1611.80	France
soybean	Itaqui	5688.83	Sines	3196.05	Hungary
soybean	Itaqui	5688.83	Sines	6137.72	Azerbaijan
soybean	Itaqui	5688.83	Sines	5927.57	Armenia
soybean	Itaqui	5688.83	Sines	3688.22	North Macedonia
soybean	Itaqui	5688.83	Sines	826.24	Gibraltar
soybean	Itaqui	5688.83	Sines	3366.39	Serbia
soybean	Itaqui	5688.83	Sines	2595.84	Germany
soybean	Itaqui	5688.83	Sines	2730.34	Slovenia
soybean	Itaqui	5688.83	Sines	2876.12	Czech Republic
soybean	Itaqui	5688.83	Sines	908.63	Morocco
soybean	Itaqui	5688.83	Sines	2553.62	Libya
soybean	Itaqui	6755.82	Tarragona	3018.03	Egypt
soybean	Itaqui	6755.82	Tarragona	980.07	Tunisia
soybean	Itaqui	6755.82	Tarragona	545.49	Algeria
soybean	Itaqui	6755.82	Tarragona	3066.99	Cyprus
soybean	Itaqui	6755.82	Tarragona	1330.87	Malta

a The table shows the distance, in kilometers, between the ports and the capital city or centroid of the destination country. Please note that differences may occur mainly in terms of flows, but also in terms of origin ports and distribution centers, between different months.

**Table 3:** Viability of the Port of Sines in the SCND for its Different Minimum Volumes (in Kton) to Justify the Grains Terminal When θ = 0.95 and Respective Costs (in Millions of Euros) of SCN, and Average Volumes (in Kton) of the Port of Sines

min Vol	avg costs	avg. vol	avg. vol. 5% worst-cases	*W*
5000	1732.039	18928.100	17782.786	1
7000	1732.046	18869.496	17675.353	1
9000	1732.031	18919.722	17693.824	1
11,000	1732.038	18956.388	18001.470	1
13,000	1732.018	18902.870	17880.494	1
15,000	1732.047	18869.518	17766.340	1
17,000	1732.041	18883.729	17777.446	1
19,000	1732.052	20036.902	19000.428	1
21,000	1731.987	22116.833	21527.575	1
22,000	1732.551	22119.046	22000.037	1
22,500	1732.389	0.000	0.000	0
23,000	1732.415	0.000	0.000	0

**Figure 8:**
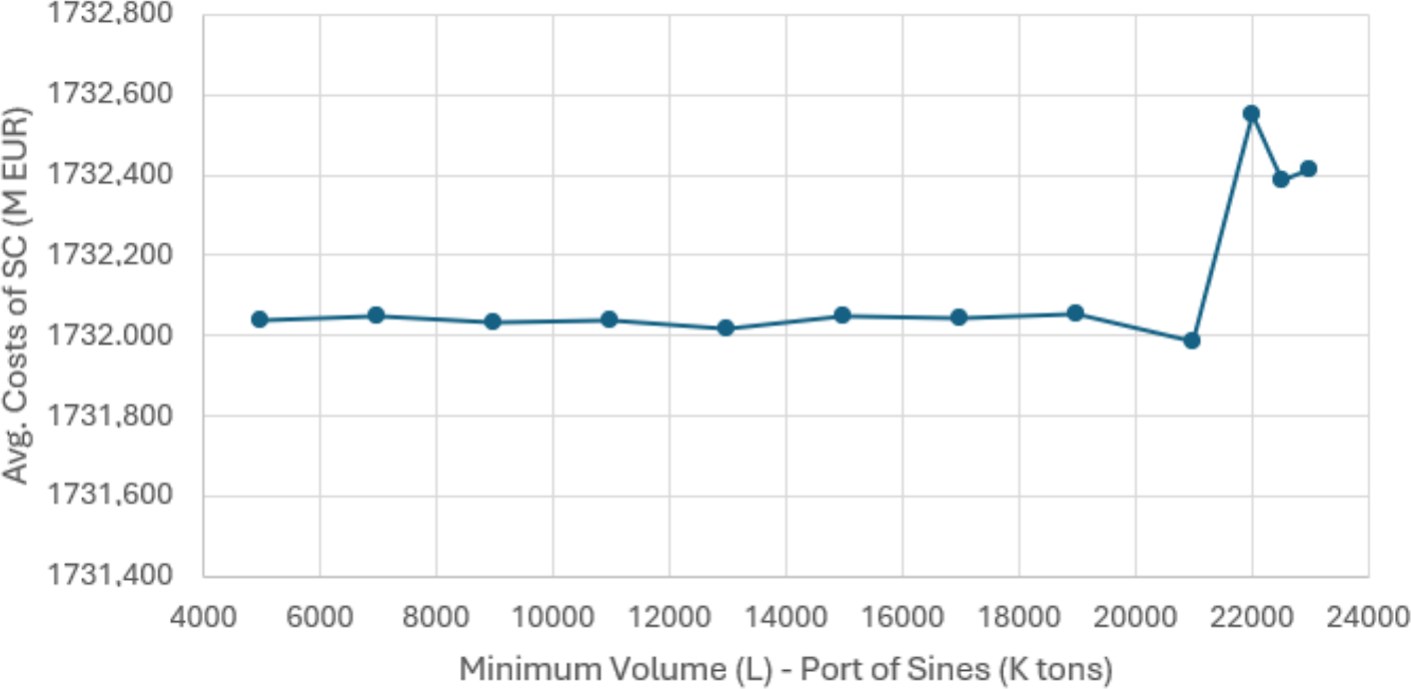
Average costs for various minimum volumes passing through the port of Sines in SC.

**Figure 9:**
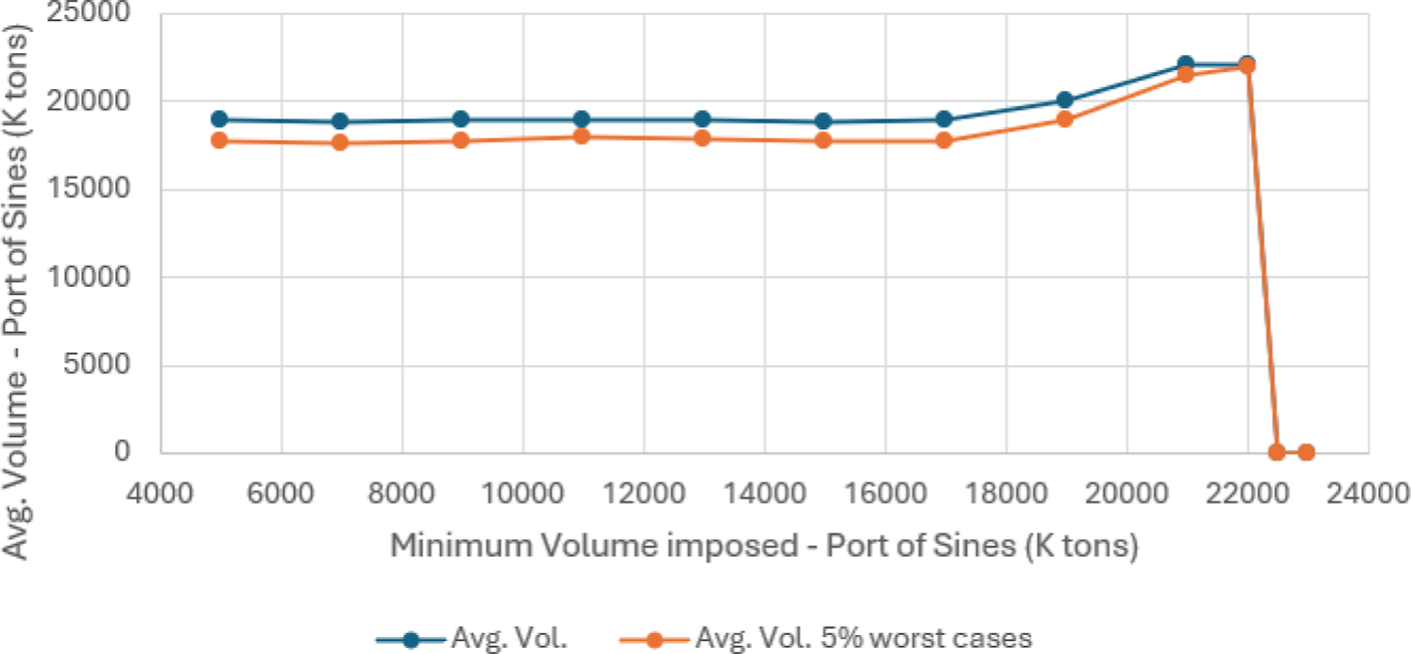
Average volumes and average 5% worst-case volumes for various minimum volumes passing through the port of Sines in SC.

**Figure 10:**
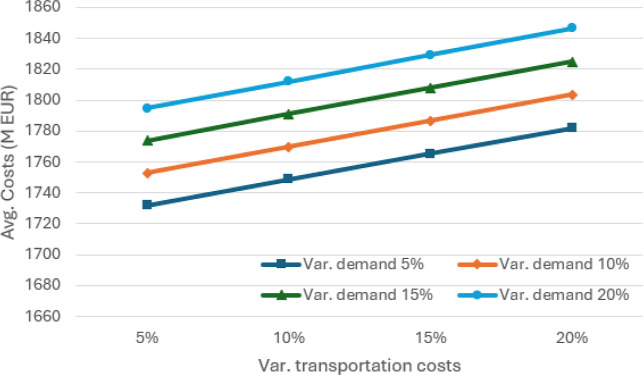
Viability of the port of Sines in the SCND for different values of absolute variations in demand and in transportation costs to justify the grains terminal when minimum volume is 5000 kton and θ = 0.95 and respective Avg costs (in millions of euros) of SCN. In all cases *W* = 1.

**11:**
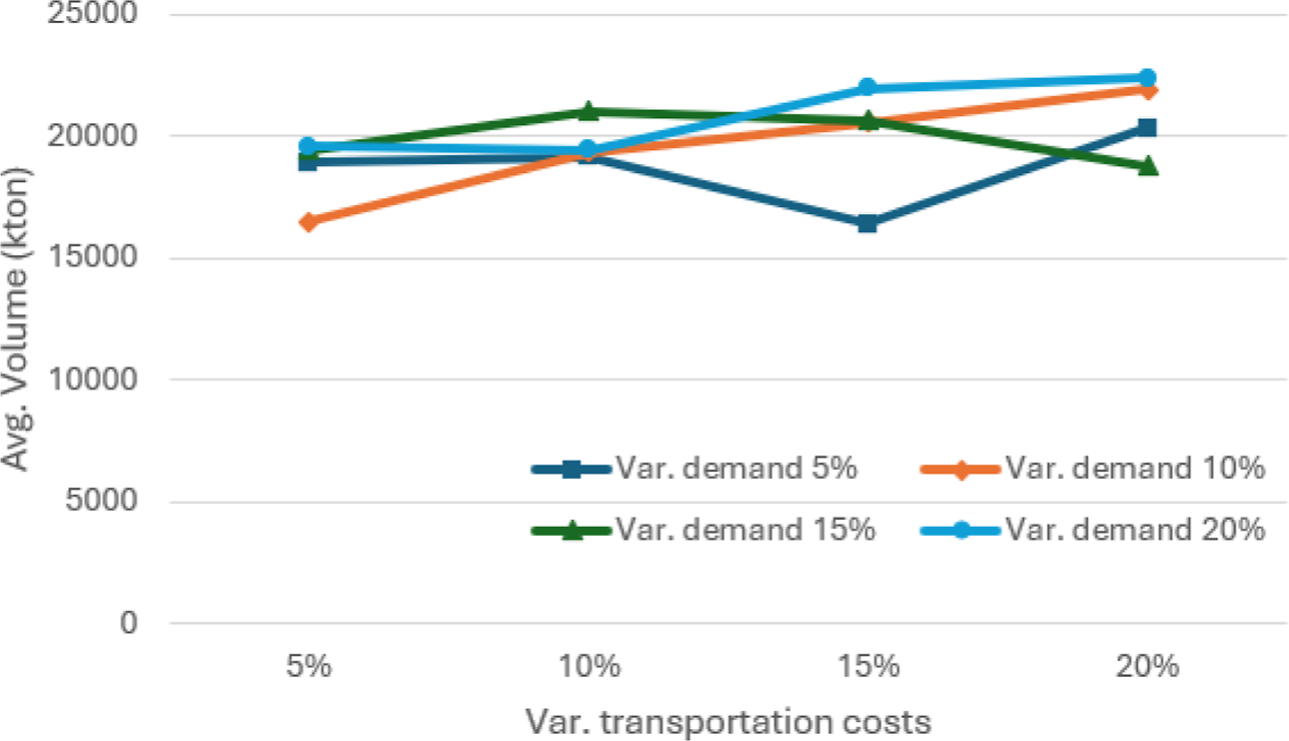
Viability of the port of sines in the SCND for different values of absolute variations in demand and in transportation costs to justify the grains terminal when minimum volume is 5000 kton and θ = 0.95 and respective average volumes (in kton) of the port of sines. In all cases *W* = 1.

#### 5.1.3 Objective Function and Constraints

The objective function (eq 1) minimizes the costs in the supply chain network to distribute the product(s) from the supply source port(s) *i* to the distribution center port(s) *k* and from distribution center port(s) *k* to the destination countries *j*, using the intermodal transportation combination modes *l*_*jk*_.

1 MinDCX+TCX+TCY+DCY

2 $$\begin{array}{l} Subject\;to:\\ \sum_{k=1}^{b}\sum_{l_{jk}=1}^{v_{jk}}Y_{jkl_{jk}p}^{t}=1,\forall j,p,t \end{array}$$

3 $$\sum_{j=1}^{m}\sum_{l_{jk}=1}^{v_{jk}}d_{jp}^{t}Y_{jkl_{jk}p}^{t}\leq e_{kp}^{t}Z_{kp}^{t},\forall k,p,t$$

4 $$\sum_{j=1}^{m}\sum_{l_{jk}=1}^{v_{jk}}d_{jp}^{t}Y_{jkl_{jk}p}^{t}=\sum_{i=1}^{n}\rho_{ip}H_{ikp}^{t},\forall k,p,t$$

5 $$H_{ikp}^{t}\leq \min\left\{g_{ip}^{t},e_{kp}^{t}\right\}X_{ikp}^{t},\forall i,k,p,t$$

6 $$\sum_{k=1}^{b}H_{ikp}^{t}\leq g_{ip}^{t},\forall i,p,t$$

7 $$\begin{array}{l} X_{ikp}^{t}\in \left\{0,1\right\},\forall i,k,p,t;Y_{jkl_{jk}p}^{t}\in \left\{0,1\right\},\forall j,k,l_{jk},p,t;Z_{kp}^{t}\in \left\{0,1\right\},\forall k,p,t;\\ H_{ikp}^{t}\geq 0,\forall i,k,p,t \end{array}$$

The meaning of each term in the objective function is briefly described below:

-
$CX=\sum_{t=1}^{ht}\sum_{p=1}^{np}\sum_{k=1}^{b}f_{kp}Z_{kp}^{t}$ , is the sum of fixed operating costs associated with the use of distribution centers *k* in the supply chain;
-
$TCX=\sum_{t=1}^{ht}\sum_{p=1}^{np}\sum_{i=1}^{n}\sum_{k=1}^{b}\left(c_{ikp}+fc_{k}\right)H_{ikp}^{t}$ , is the sum of transportation costs from the origin ports to the distribution center ports plus the sum of the costs associated with the operations in the ports (e.g., drying, fumigation, cleaning, and storage);
-
$TCY=\sum_{t=1}^{ht}\sum_{p=1}^{np}\sum_{k=1}^{b}\sum_{j=1}^{m}\sum_{l_{jk}=1}^{v_{jk}}\left(r_{jkl_{jk}}+st_{jkl_{jk}}\right)d_{jp}^{t}Y_{jkl_{jk}p}^{t}$ , is the sum of the transportation costs for the required demand from the distribution centers to the destination countries plus the sum of the entrance and exit (e.g., road, rail, sea) costs at the distribution centers *k* to the destinations *j*;
-
$DCY=\sum_{t=1}^{ht}\sum_{p=1}^{np}\sum_{i=1}^{n}\sum_{k=1}^{b}fsc_{i}X_{ikp}^{t}$ , is the sum of fixed operating costs associated with the use of the origin ports *i* in the supply chain.

The constraints (eq 2) ensure that the destination country’s demand is fully satisfied by distribution centers and mode combinations for all products and months. Each country’s demand for each product and month is met by a single distribution center. An origin can serve one or more distribution centers for each product and month. The constraints (eqs 3–5), guarantee that the capacities of the distribution centers and suppliers are not exceeded and that the demand in the countries is satisfied (eq 3); ensures that the total demand allocated to a distribution center for a given product and period does not exceed its capacity (eq 4); means that the sum of the total demand across all countries, products, and months, equals the incoming flow of the product that arrives at the distribution centers from the suppliers’ ports after the transformation process, in the case of soybean (eqs 5. and 6) secures that the supplier and transshipment port capacities are not exceeded for all products and months when the supplier and transshipment ports are used (eq 7). sets the domains of the decision variables.

The model can be improved by incorporating the following inequalities.

8 $$\sum_{i=1}^{n}H_{ikp}^{t}\leq e_{kp}^{t}Z_{kp}^{t},\forall k,p,t$$

9 $$\sum_{l_{jk}=1}^{v_{jk}}Y_{jkl_{jk}p}^{t}\leq Z_{kp}^{t},\forall j,k,p,t$$

Constraint (eq 8) ensures that supplier and transshipment port capacities are not exceeded for all products and months, provided that the supplier and transshipment ports are utilized whenever those facilities are active. Constraints (eq 9) ensure that exactly one transportation mode combination is selected from the transshipment port to the destination. Although not strictly required for model validity, their inclusion yields a tighter formulation, making the linear relaxation closer to the convex hull of the feasible solutions. Such a model can generally be solved more efficiently.

### 5.2 Stochastic Model for the Demand and Transportation Cost

This section presents a scenario-based stochastic optimization model to minimize the expected supply chain cost under demand and transportation cost uncertainty. A finite set of scenarios represents uncertainty, each with an associated probability of occurrence estimated using a frequency-based approach (prob_*s*_). Each scenario was generated by varying the demand and cost parameters around their nominal values within uncertainty ranges of [−5%, 5%] and [−20%, 20%], capturing both historical and seasonal fluctuations in transport and demand conditions. To assess the feasibility of including the Sines port in the supply chain, a conditional value-at-risk (CVaR) constraint is imposed on the flow of goods through this port. Given that *VaR*[*X*, *q*] = *F* ^–1^(*q*) is the quantile *q* ∈ (0, 1) of the distribution *F* of the random variable *X*, *CVaR*[*X*, *q*] = *E*[*X*|*X* > *F* ^–1^(*q*)]. *VaR*[*X*, *q*] is the value-at-risk for quantile *q*, and *CVaR*[*X*, *q*] is the corresponding conditional value-at-risk. This constraint ensures that the port is activated only when the average volume of goods in the worst-case θ% scenarios exceeds a minimum threshold that justifies its economic viability. A new binary variable *W* is introduced to indicate whether the Sines port (which is denoted by k*) will be operating or not, as well as a continuous non-negative variable, *VAR* (value-at-risk), computed by the model. The remaining decision variables mirror those used in the deterministic model, with an additional index *s* that indexes the different scenario-specific realizations.

This means that all those variables are adjustable to the uncertain parameters.

$$\begin{array}{l} \mathrm{Min}\,\;COST=\overset{ns}{\underset{s=1}{\sum }}\overset{ht}{\underset{t=1}{\sum }}\overset{np}{\underset{p=1}{\sum }}\overset{b}{\underset{k=1}{\sum }}f_{kp}Z_{kps}^{t}prob_{s}+\overset{ns}{\underset{s=1}{\sum }}\overset{ht}{\underset{t=1}{\sum }}\overset{np}{\underset{p=1}{\sum }}\overset{n}{\underset{i=1}{\sum }}\overset{b}{\underset{k=1}{\sum }}\left(c_{ikps}+fc_{ks}\right)H_{ikps}^{t}prob_{s}+\\ \overset{ns}{\underset{s=1}{\sum }}\overset{ht}{\underset{t=1}{\sum }}\overset{np}{\underset{p=1}{\sum }}\overset{b}{\underset{k=1}{\sum }}\overset{m}{\underset{j=1}{\sum }}\overset{v_{jk}}{\underset{l_{jk}=1}{\sum }}\left(r_{jkl_{jk}s}+st_{jkl_{jk}s}\right)d_{jps}^{t}Y_{jkl_{jk}ps}^{t}prob_{s}+\\ \overset{ns}{\underset{s=1}{\sum }}\overset{ht}{\underset{t=1}{\sum }}\overset{np}{\underset{p=1}{\sum }}\overset{n}{\underset{i=1}{\sum }}\overset{b}{\underset{k=1}{\sum }}fsc_{i}X_{ikps}^{t}prob_{s} \end{array}$$ 10

11 $$\begin{array}{l} Subject\;to:\\ VAR+\frac{1}{1-\theta }\sum_{s=1}^{ns}DV_{s}prob_{s}\leq U-L+M\left(1-W\right) \end{array}$$

12 $$DV_{s}\geq U-\sum_{t=1}^{ht}\sum_{p=1}^{np}\sum_{i=1}^{n}H_{ik^{*}ps}^{t}-VAR,\forall s$$

13 $$\sum_{k=1}^{b}\sum_{l_{jk}=1}^{v_{jk}}Y_{jkl_{jk}ps}^{t}=1,\forall j,p,t,s$$

14 $$\sum_{j=1}^{m}\sum_{l_{jk}=1}^{v_{jk}}d_{jps}^{t}Y_{jkl_{jk}ps}^{t}\leq e_{kps}^{t}Z_{kps}^{t},\forall k,p,t,s$$

15 $$\sum_{j=1}^{m}\sum_{l_{jk}=1}^{v_{jk}}d_{jps}^{t}Y_{jkl_{jk}ps}^{t}=\sum_{i=1}^{n}\rho_{ip}H_{ikps}^{t},\forall k,p,t,s$$

16 $$H_{ikps}^{t}\leq \min\left\{g_{ips}^{t},e_{kps}^{t}\right\}X_{ikps}^{t},\forall i,k,p,t,s$$

17 $$\sum_{i=1}^{n}H_{ikps}^{t}\leq e_{kps}^{t}Z_{kps}^{t},\forall k,p,t,s$$

18 $$\sum_{l_{jk}=1}^{v_{jk}}Y_{jkl_{jk}ps}^{t}\leq Z_{kps}^{t},\forall j,k,p,t,s$$

19 $$\sum_{k=1}^{b}H_{ikps}^{t}\leq g_{ips}^{t},\forall i,p,t,s$$

20 $$Z_{k^{*}ps}^{t}\leq W,\forall p,t,s$$

21 $$\begin{array}{l} X_{ikps}^{t}\in \left\{0,1\right\},\forall i,k,p,t,s;Y_{jkl_{jk}ps}^{t}\in \left\{0,1\right\},\forall j,k,l_{jk},p,t,s;Z_{kps}^{t}\in \left\{0,1\right\},\forall k,p,t,s;\\ H_{ikps}^{t}\geq 0,\forall i,k,p,t,s;W\in \left\{0,1\right\};VAR\geq 0 \end{array}$$

The objective function, eq 10, and associated constraints, except (eqs 11 and 12), have the same meaning as the corresponding ones of the deterministic model, with the necessary extension to the case of existing scenarios. Constraints (eqs 11 and 12) model the CVaR imposed on the annual volume transported through the Sines port, whose index is denoted by k*. In our case, a lower bound *L* was imposed on this volume to ensure the economic viability of the port. However, for technical reasons,^34^ the CVaR constraint must be stated as an upper bound constraint. Hence, instead of imposing *L* on the volume, an upper bound was imposed on the port capacity that is not used, $=\sum_{t=1}^{ht}\sum_{p=1}^{np}e_{k^{*}p}^{t}$ . Consequently, the flow passing through the port in scenario *s*, is defined as

$$V\left(s\right)=\sum_{t=1}^{ht}\sum_{p=1}^{np}\sum_{i=1}^{n}H_{ik^{*}ps}^{t}$$

The capacity of the port that is not used in scenario *s* is *U*–*V*(*s*). The CVaR constraint (eq 11) can be written as follows

$$VAR+\frac{1}{1-\theta }\sum_{s=1}^{ns}\max\left\{0,\left(U-V\left(s\right)-VAR\right)\right\}prob_{s}\leq U-L$$

To linearize this constraint, deviation variables, *DV*_*s*_, were introduced in the model

$$DV_{s}=\max\left\{0,\left(U-V\left(s\right)-VAR\right)\right\}$$

This expression is ensured with constraints (eq 12). Finally, the term *M*(1 – *W*) must be added to the right side of the (eq 11) constraint to ensure that the constraint is redundant when *W* = 0, where *M* is a large number (eq 20). enforces that the W value equals 1 when the Sines port operates as a distribution center, justified by CVaR constraints.

### 5.3 Biobjective Model with CO_2_ Equivalent Emissions

To address growing concerns about reducing greenhouse gas emissions and mitigating global warming, CO_2_-equivalent emissions have been incorporated into the derivation of a model that reflects sustainability concerns. CO_2_-equivalent emissions refers to emissions of CO_2_, CH_4_, and N_2_O. Although equivalent emissions are considered in most academic studies, they may not yet be the most critical factor for decision-makers, who remain focused on satisfying demand at the lowest cost while possibly considering environmentally friendly options to do so. Therefore, a biobjective problem is considered by adding a second objective function dedicated to minimizing the expected CO_2_ equivalent emissions (eq 22)­

22 $$CO2=\sum_{s=1}^{ns}\sum_{t=1}^{ht}\sum_{p=1}^{np}\sum_{i=1}^{n}\sum_{k=1}^{b}ex_{ik}H_{ikps}^{t}prob_{s}+\sum_{s=1}^{ns}\sum_{t=1}^{ht}\sum_{p=1}^{np}\sum_{k=1}^{b}\sum_{j=1}^{m}\sum_{l_{jk}=1}^{v_{jk}}ey_{jkl_{jk}}d_{jps}^{t}Y_{jkl_{jk}ps}^{t}prob_{s}$$

The epsilon-constrained method^35^ is used to solve the biobjective problem by minimizing (eq 22) and imposing a constraint on the expected cost COST ≤ (1 + ϵ)z* where z* is the optimal expected cost of the single objective function problem ([Disp-formula eq10]–[Disp-formula eq21]).

**Figure 12:**
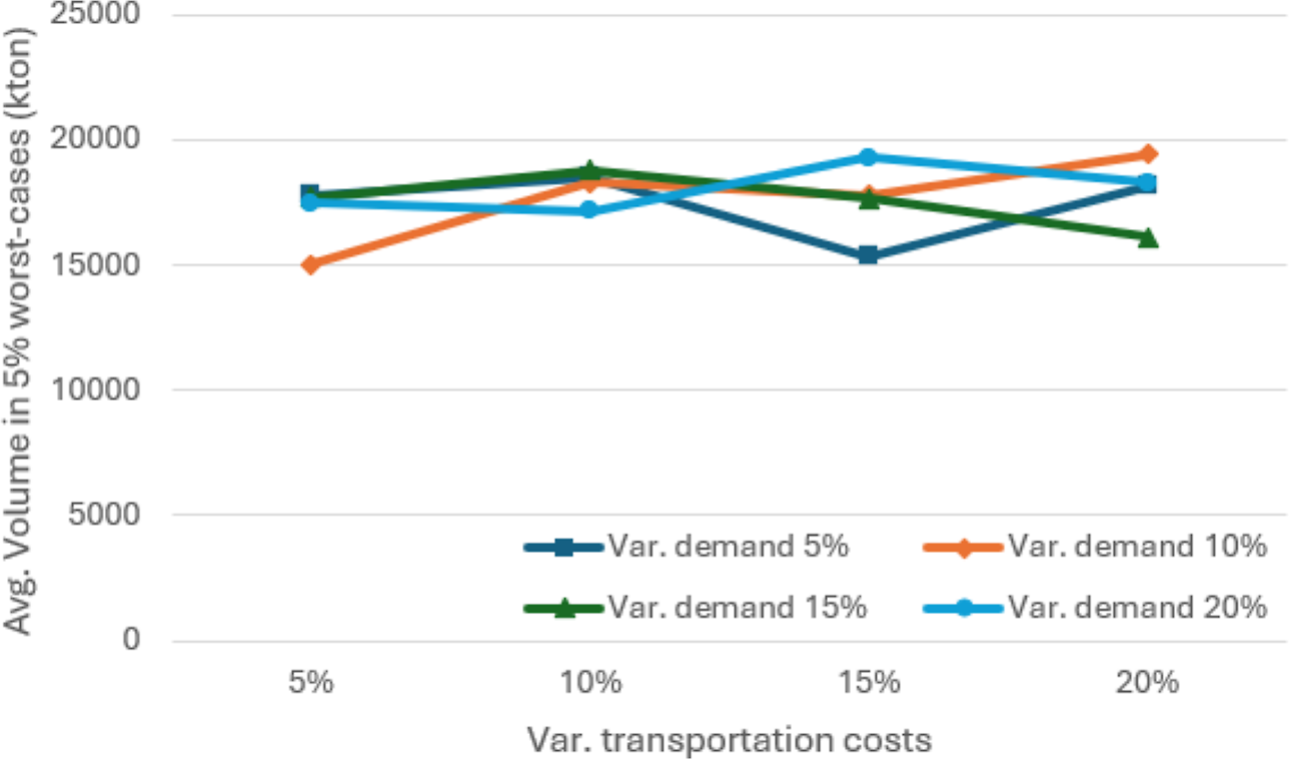
Viability of the port of Sines in the SCND for different values of absolute variations in demand and in transportation costs to justify the grains terminal when minimum volume is 5000 kton and θ = 0.95 and respective average volumes of 5% worst-cases (in kton) of the port of Sines. In all cases *W* = 1.

**Table 4:** Bi-Objective Stochastic Model Solution Minimizes CO_2_-Equivalent Emissions for the Corn Scenario 1 and Month 12 of SCN^a^

product	origin	distance(km)	distribution center	distance(km)	destination
corn	New Orleans	8896.37	Rotterdam	941.86	Liechtenstein
corn	New Orleans	8896.37	Rotterdam	2153.83	Moldova
corn	New Orleans	8896.37	Rotterdam	2168.73	Estonia
corn	New Orleans	8896.37	Rotterdam	110.17	Netherlands
corn	New Orleans	8896.37	Rotterdam	2501.07	Russia
corn	New Orleans	8896.37	Rotterdam	2186.10	Norway
corn	New Orleans	8896.37	Rotterdam	817.87	Denmark
corn	New Orleans	8896.37	Rotterdam	1701.08	Lithuania
corn	New Orleans	8896.37	Rotterdam	1131.52	Poland
corn	New Orleans	8896.37	Rotterdam	1466.76	San Marino
corn	New Orleans	8896.37	Rotterdam	2240.57	Ukraine
corn	New Orleans	8896.37	Rotterdam	1910.03	Latvia
corn	New Orleans	8896.37	Rotterdam	2846.48	Finland
corn	New Orleans	8896.37	Rotterdam	1827.63	Belarus
corn	New Orleans	8896.37	Rotterdam	4120.31	Georgia
corn	New Orleans	8896.37	Rotterdam	2071.24	Sweden
corn	New Orleans	8896.37	Rotterdam	4365.98	Azerbaijan
corn	New Orleans	8896.37	Rotterdam	4292.54	Armenia
corn	New Orleans	8896.37	Rotterdam	2492.12	Gibraltar
corn	New Orleans	8896.37	Rotterdam	538.40	Germany
corn	New Orleans	8896.37	Rotterdam	1019.71	Czech Republic
corn	New Orleans	8841.87	Antwerp	734.66	Switzerland
corn	New Orleans	8841.87	Antwerp	2149.82	Albania
corn	New Orleans	8841.87	Antwerp	1071.97	Austria
corn	New Orleans	8841.87	Antwerp	76.81	Belgium
corn	New Orleans	8841.87	Antwerp	264.41	Luxembourg
corn	New Orleans	8841.87	Antwerp	2043.74	Romania
corn	New Orleans	8841.87	Antwerp	2353.26	Bulgaria
corn	New Orleans	8841.87	Antwerp	1645.08	Bosnia and Herzegovina
corn	New Orleans	8841.87	Antwerp	1992.78	Montenegro
corn	New Orleans	8841.87	Antwerp	1493.58	Slovakia
corn	New Orleans	8841.87	Antwerp	1450.01	Croatia
corn	New Orleans	8841.87	Antwerp	1461.02	Hungary
corn	New Orleans	8841.87	Antwerp	1886.22	Serbia
corn	New Orleans	8841.87	Antwerp	1229.72	Slovenia
corn	New Orleans	8057.41	Lisbon	642.69	Spain
corn	New Orleans	8528.55	Le Havre	784.32	United Kingdom
corn	New Orleans	8528.55	Le Havre	468.31	France
corn	New Orleans	8528.55	Le Havre	2548.67	Iceland
corn	New Orleans	8528.55	Le Havre	1019.80	Ireland
corn	Itaqui	5688.83	Sines	280.90	Portugal
corn	Itaqui	5718.43	Lisbon	642.69	Spain
corn	Itaqui	6755.82	Tarragona	1106.47	Italy
corn	Itaqui	5657.02	Casablanca	2327.74	Monaco
corn	Itaqui	5657.02	Casablanca	4928.72	Turkey
corn	Itaqui	5657.02	Casablanca	1675.58	Andorra
corn	Itaqui	5657.02	Casablanca	3376.93	Greece
corn	Itaqui	5657.02	Casablanca	3720.76	North Macedonia
corn	Itaqui	5657.02	Casablanca	393.78	Morocco
corn	Itaqui	5657.02	Casablanca	3925.28	Egypt
corn	Itaqui	5657.02	Casablanca	1864.30	Tunisia
corn	Itaqui	5657.02	Casablanca	1190.74	Algeria
corn	Itaqui	5657.02	Casablanca	2431.74	Libya
corn	Itaqui	5657.02	Casablanca	3977.89	Cyprus
corn	Itaqui	5657.02	Casablanca	2238.11	Malta
corn	Santos	7967.53	Casablanca	2327.74	Monaco
corn	Santos	7967.53	Casablanca	4928.72	Turkey
corn	Santos	7967.53	Casablanca	1675.58	Andorra
corn	Santos	7967.53	Casablanca	3376.93	Greece
corn	Santos	7967.53	Casablanca	3720.76	North Macedonia
corn	Santos	7967.53	Casablanca	393.78	Morocco
corn	Santos	7967.53	Casablanca	3925.28	Egypt
corn	Santos	7967.53	Casablanca	1864.30	Tunisia
corn	Santos	7967.53	Casablanca	1190.74	Algeria
corn	Santos	7967.53	Casablanca	2431.74	Libya
corn	Santos	7967.53	Casablanca	3977.89	Cyprus
corn	Santos	7967.53	Casablanca	2238.11	Malta

a Include the distances, in kilometers, between the ports and the capital city or centroid of the destination country. Please note that differences may occur mainly in the flows, but also in terms of origin ports and distribution centers, between different months.

**Table 5:** Bi-Objective Stochastic Model Solution Minimizes CO_2_-Equivalent Emissions for the Soybean Scenario 1 and Month 12 of SCN^a^

product	origin	distance(km)	distribution center	distance(km)	destination
soybean	New Orleans	8896.37	Rotterdam	2153.83	Moldova
soybean	New Orleans	8896.37	Rotterdam	2168.73	Estonia
soybean	New Orleans	8896.37	Rotterdam	110.17	Netherlands
soybean	New Orleans	8896.37	Rotterdam	2501.07	Russia
soybean	New Orleans	8896.37	Rotterdam	2186.10	Norway
soybean	New Orleans	8896.37	Rotterdam	817.87	Denmark
soybean	New Orleans	8896.37	Rotterdam	2240.57	Ukraine
soybean	New Orleans	8896.37	Rotterdam	1910.03	Latvia
soybean	New Orleans	8896.37	Rotterdam	2846.48	Finland
soybean	New Orleans	8896.37	Rotterdam	1827.63	Belarus
soybean	New Orleans	8896.37	Rotterdam	2071.24	Sweden
soybean	New Orleans	8896.37	Rotterdam	538.40	Germany
soybean	New Orleans	8896.37	Rotterdam	1019.71	Czech Republic
soybean	New Orleans	8841.87	Antwerp	734.66	Switzerland
soybean	New Orleans	8841.87	Antwerp	76.81	Belgium
soybean	New Orleans	8841.87	Antwerp	264.41	Luxembourg
soybean	New Orleans	8841.87	Antwerp	1493.58	Slovakia
soybean	New Orleans	8528.55	Le Havre	2166.79	Lithuania
soybean	New Orleans	8528.55	Le Havre	1597.22	Poland
soybean	New Orleans	8528.55	Le Havre	784.32	United Kingdom
soybean	Itaqui	5688.83	Sines	2316.39	Liechtenstein
soybean	Itaqui	5688.83	Sines	2496.31	San Marino
soybean	Itaqui	5688.83	Sines	280.90	Portugal
soybean	Itaqui	5688.83	Sines	702.52	Spain
soybean	Itaqui	5688.83	Sines	1611.8	France
soybean	Itaqui	5688.83	Sines	826.24	Gibraltar
soybean	Itaqui	5718.43	Lisbon	3134.81	Iceland
soybean	Itaqui	5718.43	Lisbon	1965.98	Ireland
soybean	Itaqui	7201.65	Le Havre	2166.79	Lithuania
soybean	Itaqui	7201.65	Le Havre	1597.22	Poland
soybean	Itaqui	7201.65	Le Havre	784.32	United Kingdom
soybean	Itaqui	5657.02	Casablanca	2327.74	Monaco
soybean	Itaqui	5657.02	Casablanca	2317.29	Italy
soybean	Itaqui	5657.02	Casablanca	3778.70	Albania
soybean	Itaqui	5657.02	Casablanca	3080.42	Austria
soybean	Itaqui	5657.02	Casablanca	3954.63	Romania
soybean	Itaqui	5657.02	Casablanca	4009.30	Bulgaria
soybean	Itaqui	5657.02	Casablanca	4928.72	Turkey
soybean	Itaqui	5657.02	Casablanca	1675.58	Andorra
soybean	Itaqui	5657.02	Casablanca	3301.12	Bosnia and Herzegovina
soybean	Itaqui	5657.02	Casablanca	3646.71	Montenegro
soybean	Itaqui	5657.02	Casablanca	5665.68	Georgia
soybean	Itaqui	5657.02	Casablanca	3376.93	Greece
soybean	Itaqui	5657.02	Casablanca	3106.04	Croatia
soybean	Itaqui	5657.02	Casablanca	3371.92	Hungary
soybean	Itaqui	5657.02	Casablanca	6117.18	Azerbaijan
soybean	Itaqui	5657.02	Casablanca	5907.03	Armenia
soybean	Itaqui	5657.02	Casablanca	3720.76	North Macedonia
soybean	Itaqui	5657.02	Casablanca	3542.25	Serbia
soybean	Itaqui	5657.02	Casablanca	2906.20	Slovenia
soybean	Itaqui	5657.02	Casablanca	393.78	Morocco
soybean	Itaqui	5657.02	Casablanca	3925.28	Egypt
soybean	Itaqui	5657.02	Casablanca	1864.30	Tunisia
soybean	Itaqui	5657.02	Casablanca	1190.74	Algeria
soybean	Itaqui	5657.02	Casablanca	2431.74	Libya
soybean	Itaqui	5657.02	Casablanca	3977.89	Cyprus
soybean	Itaqui	5657.02	Casablanca	2238.11	Malta
soybean	Santos	7967.53	Casablanca	2327.74	Monaco
soybean	Santos	7967.53	Casablanca	2317.29	Italy
soybean	Santos	7967.53	Casablanca	3778.70	Albania
soybean	Santos	7967.53	Casablanca	3080.42	Austria
soybean	Santos	7967.53	Casablanca	3954.63	Romania
soybean	Santos	7967.53	Casablanca	4009.30	Bulgaria
soybean	Santos	7967.53	Casablanca	4928.72	Turkey
soybean	Santos	7967.53	Casablanca	1675.58	Andorra
soybean	Santos	7967.53	Casablanca	3301.12	Bosnia and Herzegovina
soybean	Santos	7967.53	Casablanca	3646.71	Montenegro
soybean	Santos	7967.53	Casablanca	5665.68	Georgia
soybean	Santos	7967.53	Casablanca	3376.93	Greece
soybean	Santos	7967.53	Casablanca	3106.04	Croatia
soybean	Santos	7967.53	Casablanca	3371.92	Hungary
soybean	Santos	7967.53	Casablanca	6117.18	Azerbaijan
soybean	Santos	7967.53	Casablanca	5907.03	Armenia
soybean	Santos	7967.53	Casablanca	3720.76	North Macedonia
soybean	Santos	7967.53	Casablanca	3542.25	Serbia
soybean	Santos	7967.53	Casablanca	2906.20	Slovenia
soybean	Santos	7967.53	Casablanca	393.78	Morocco
soybean	Santos	7967.53	Casablanca	3925.28	Egypt
soybean	Santos	7967.53	Casablanca	1864.30	Tunisia
soybean	Santos	7967.53	Casablanca	1190.74	Algeria
soybean	Santos	7967.53	Casablanca	2431.74	Libya
soybean	Santos	7967.53	Casablanca	3977.89	Cyprus
soybean	Santos	7967.53	Casablanca	2238.11	Malta
soybean	Montevideo	11215.21	Le Havre	2166.79	Lithuania
soybean	Montevideo	11215.21	Le Havre	1597.22	Poland
soybean	Montevideo	11215.21	Le Havre	784.32	United Kingdom

a Include the distances, in kilometers, between the ports and the capital city or centroid of the destination country. Please note that differences may occur mainly in the flows, but also in terms of origin ports and distribution centers, between different months.

**Table 6:** Viability of the Port of Sines in the SCND for Different Values of Variations of ? in Average Costs to Justify the Grains Terminal When Minimum Volume is 5000 kton and θ = 0.95 and Respective CO_2_ Equivalent Emissions (in Millions of Tons) of SCN, and Average Volumes (in Kton) of the Port of Sines^a^

var.ϵ costs	avg.CO_2_-eq	avg. vol	avg. vol. 5% worst-cases	*W*
10%	15.123	8339.009	8142.198	1
10%	15.129	0.000	0.000	0
15%	15.123	8339.009	8142.198	1
15%	15.129	0.000	0.000	0

a The epsilon-constrained method was applied with variations of 10% and 15% in the COST function value. The value *W* = 0 is imposed to compare the results of the solutions.

## 6 Results and Discussion

This section conducts three complementary analyses of the supply chain and evaluates the relevance of the port of Sines within it. The first study, described in Section 6.2, considers transportation costs only, while the second study, reported in Section 6.3, incorporates CO_2_ emissions.

The results were obtained using a personal computer with an AMD Ryzen 7 5800H processor and 16 GB of RAM. The Gurobi optimizer, implemented in Python, was utilized, along with the NetworkX and Geopandas libraries.

### 6.1 Transportation Costs of SCND

The full SCND model was evaluated by minimizing the expected cost over 145 scenarios: the nominal scenario and the Cartesian product of two independent sets of 12 random scenarios (from two independent uniform distributions), each with a given percentage change of demand and transportation costs. Equations (eqs 11, 12 and 20) of the formulation in subsection [Sec sec5.2] were not included.

The results of the sensitivity analysis in terms of percentage absolute variation are illustrated in Figures 5–7. As expected, variations in demand and transportation costs increase the average expected costs of SCND for distributing soybean and corn in Europe and North Africa. Sines emerges as one of the main players in this SC, handling an average of more than 17,700 kton of soybean and corn and more than 16,400 kton in the 5% worst cases, when considering absolute variations between 5% and 20%.

Tables 1 and 2 represents a SCND solution for scenario 1 and month 12, with the following origin ports: Itaqui, Santos, and New Orleans. The transshipment ports involved in the distribution process include Lisbon, Sines, Le Havre, Gijón, Casablanca, Tarragona, Antwerp, and Rotterdam. The strategic value of the port of Sines is evident from its central role in this SC. Advantages include its strategic location, access to cost-effective rail infrastructure, and the ability to supply several locations in Southern Europe. It can also extend operations to more locations using Handy-size vessels, which are more cost-effective than terrestrial transport. In contrast, Lisbon is the nearest Portuguese port, but its SC processes are less efficient than those at Sines. Sines is also a deep-water port, and its natural conditions enable it to easily receive Panamax and larger vessels, mainly from South America (e.g., the Port of Itaqui) and from North America (e.g., the Port of New Orleans). However, this study focuses on assessing the role of the Port of Sines within the supply chain network, rather than comparing its performance with that of other ports. `

### 6.2 Transportation Costs Focused on the Port of Sines

It is important to note that the design of the supply chain typically involves different players. It is assumed that each player follows a minimum cost policy. However, for the port of Sines, the decision is made by other players. That is why the decision to open this port was modeled as a constraint, with no cost associated with this decision in the objective function. However, it should be noted that the decision of using or not using the port is taken by the model, which may not lead to a real solution because, if the port is open, the players are not forced to respect the minimum volume passing through the port for the economic viability of the grains terminal. Hence, three situations may occur: (i) the case where the model selects the port and the CVaR constraint is not active; this case means that the port should be open, since the expected flow in the worst θ case is above the established minimum; (ii) the case where the port is open and the CVaR constraint is active; this is the case where the actual flow may fall below the minimum threshold, and the minimum value may not be satisfied; (iii) the case where the port is not open; this clearly indicates that, for the minimum values established, the port is not viable. Table 3 provides the results obtained for different minimum values.

As shown in Table 3, the corresponding solution values for the economic viability limit of the port of Sines (5000 kton of soybean and corn) yield an average total volume exceeding this minimum, indicating the port of Sines’ potential economic viability. The maximum minimum volume for the port of Sines to remain viable under the conditions in Table 3, is approximately 22,000 kton. Increasing the minimum volume for viable routes from a certain point (situations (ii) and (iii)) to a level greater than the average volume, without using equations (eqs 11, 12), and (eq 20), or choosing alternative routes (*W* = 0), increases the average cost of SCN, as well as the average quantities passing through Sines. The three situations are illustrated in Figures 8 and 9.

Figures 10–12 illustrate the sensitivity of absolute variations (both positive and negative) in demand volumes and transportation costs. In general, average costs increase as demand exhibits greater variation. The average volumes reach a minimum of 16,400 kton of soybean and corn and more than 15,000 kton in the 5% worst cases, when considering absolute variations between 5% and 20%. Although average costs tend to rise in line with variations in transportation costs, they differ slightly from those shown in Figure 5, where a minimum volume of 5000 kton is required at the Port of Sines. As can be seen in Figure 8, this occurs because the model can supply this volume with no additional effort.

### 6.3 Integrating Costs and CO_2_ Emissions

As mentioned before, growing focus on global warming has led us to consider more environmentally friendly solutions, such as those obtained by minimizing CO_2_ emissions. Therefore, the reformulated optimization problem also minimizes the average CO_2_-equivalent emissions in the transport modes used by the supply chain, while imposing a maximum average cost equal to the minimum average cost of the model presented in subsection [Sec sec5.2] considering θ = 0.95, and a minimum volume through the port of Sines (L) of 5000 kton, plus a percentage of this average cost (see subsection [Sec sec5.3]). We have considered an increase of 10% and 15%, which are deemed reasonable and allow the extraction of useful information on the role of the Port of Sines.

Tables 4 and 5 represent a SCND solution for scenario 1 and month 12, that was modified to minimize *CO*_2_-equivalent emissions when compared to Tables 1 and 2, considering paths with lower emissions and a slight increase in average costs.

In the first set of tables, where the objective is to minimize costs, the model most often chose the port of New Orleans in North America as the origin port for distributing corn and the distribution centers of Rotterdam, Antwerp, and Lisbon. However, it also chooses the origin ports of Itaqui and Santos to a lesser extent, with the main distribution centers being Sines, Antwerp, Rotterdam, Tarragona, and Casablanca. When the objective was to minimize CO_2_-equivalent emissions, the model selected the same origin ports and, in a few cases, Montevideo, but it slightly more often chose the other ports. The main distribution centers were Rotterdam, Antwerp, and Casablanca.

When considering soybean distribution, the cost-minimization results indicate New Orleans and Itaqui as the primary origin ports. The main distribution centers are Sines, Tarragona, and Antwerp, with Le Havre and Rotterdam to a lesser extent. If the minimization of CO_2_-equivalent emissions is also considered, the model selects Itaqui and Santos more often as the origin port and Casablanca (more often), Antwerp, Rotterdam, and Sines as the main distribution centers. When the second objective of minimizing greenhouse gas emissions is considered, Sines is involved in fewer distribution paths for both products.

As shown in Table 6, the results remain unchanged when using the port of Sines (*W* = 1) or not (*W* = 0), as the percentage increase in average SC costs increases. However, average CO_2_-equivalent emissions increase slightly when the port of Sines is not used (*W* = 0), from 15,123 M tons to 15,129 M tons.

This last result is another indication of the important role played by the port of Sines in this SC. The results demonstrate that, on average, solutions to the SC that consider the port of Sines are more environmentally friendly.

The overall transportation costs of a supply chain depend on the types of transportation used, which have different emissions and economic costs. Few supply chain processes have as significant an influence on business performance as transportation choices. Transportation ensures the timely movement of goods from their point of origin to their destinations, acting as the backbone of cargo flow. Given its critical role in ensuring operational efficiency and customer satisfaction, transportation must be a central element in any supply chain management strategy. As a core activity, transportation accounts for substantial economic costs and environmental impacts. Therefore, strategic decisions regarding transportation modes and logistics planning are essential for building a resilient, cost-effective, and sustainable supply chain.

## 7 Conclusions

The growth of South America’s geographic importance in food production over recent decades, driven primarily by Brazil and Argentina’s favorable agricultural conditions, has reshaped the map, particularly in the distribution of bulk soybean and corn grain. This new situation motivated the study of the potential interest of using the port of Sines as an entry point for grain imports, and to assess its role in improving the total efficiency of the SCN in distributing cargo along the markets of the South Mediterranean and MENA region (Middle East and North Africa), enhancing the resilience of the SCN. More specifically, we analyze the flow in an intermodal supply chain network (SCN) for supplying soybean and corn to Europe and North Africa, considering the shift in the production and supply of these commodities from North America to South America. Sines’ proximity to Brazil and other South American grain producers positions it as a competitive alternative to traditional European grain hubs, facilitating efficient transshipment to Mediterranean destinations and strengthening food security in the region.

Stochastic programming analysis demonstrated the economic viability of a new grain terminal in the port of Sines. On average, approximately 17,000 kt can be distributed via the flow through the Sines port to Europe and North Africa. Sensitivity analyses based on stochastic model scenarios to minimize transportation costs also confirm the economic viability of the Port of Sines, even under potential 20% changes in demand or transportation costs, and in the worst-case average of 5% scenarios.

A grain terminal at the port of Sines would be economically viable and could have a positive economic and social impact on the Portuguese region. For example, it could increase agro-industrial activity and employment opportunities. At the same time, it would ensure that soybean and corn reach their destinations in Europe and North Africa quickly and in the best possible conditions, while keeping transportation costs to a minimum. Furthermore, our results demonstrate that solutions incorporating the port of Sines are more environmentally friendly and can reduce CO_2_-equivalent emissions.

In summary, there is considerable potential to optimize grain SCN flow, with the port of Sines playing a major role as a transshipment hub for southern European countries, thereby linking new logistics partnerships and agreements to strengthen southeast European agri-food trade corridors.

## Supplementary Material



## Notes

This work is dedicated to the memory of Professor Pedro Castro. Pedro was a dedicated educator and researcher. His intelligence, stamina, motivation, and values are an inspiration to all of us. His regular presence in PSE meetings, insightful contributions, and good disposition will be deeply missed.
