# Supplementary material for: A Biobjective Stochastic Model for Intermodal Supply Chains: Application to the Corn and Soybean Flows
Source: Ind Eng Chem Res. 2026 Feb 19;65(8):4451–70. doi: 10.1021/acs.iecr.5c02623 (PMC12964394; doi:10.1021/acs.iecr.5c02623)
Supplement: Supplementary file 1 [file ie5c02623_si_001.pdf]

Supplementary Information to:

A Bi-objective Stochastic Model for Intermodal Supply Chains: Application to the Corn and Soybean Flows

Marco Marto,[1] Valentina Chkoniya,[2,3] Eduardo B. Couto,[4] Telmo Pinto,[5] Agostinho Agra,[6] and Marco S. Reis[*,7]

[1] *University of Aveiro, Aveiro Institute of Accounting and Administration and CIDMA - Center for Research & Development in Mathematics and Applications, 3810-500 Aveiro, Portugal*

[2] *University of Aveiro, Aveiro Institute of Accounting and Administration, 3810-500 Aveiro, Portugal*

[3] *University of Aveiro, GOVCOPP - Research Unit in Governance, Competitiveness and Public Policies, 3810-193 Aveiro, Portugal*

[4] *University of Aveiro, Department of Economics, Management, Industrial Engineering and Tourism, 3810-193 Aveiro, Portugal*

[5] *ALGORITMI Research Centre / LASI, University of Minho, Campus de Gualtar, 4710-057 Braga, Portugal*

[6] *Departamento de Ciências Matemáticas and Centro de Estudos Matemáticos (CEMS.UL), Faculdade de Ciências, Universidade de Lisboa, Portugal.*

[7] *University of Coimbra, CERES, Department of Chemical Engineering, 3030-790 Coimbra, Portugal*

* E-mail: marco@eq.uc.pt

Tables S1-S3 present the metadata used for establishing or computing the fixed costs, capacities, variable costs, and greenhouse gas emission parameters in the models described

in the manuscript. This metadata information includes the description of the parameters, calculation steps, units, and sources.

Table S1: Metadata for calculation of parameters part 1 of 3.

**Parameters**	**Meaning**	**Value or Calculation**	**Unit**	**Sources**
Fixed costs:				
fsc_i	fixed cost for using a supply port i	5685.00	EUR	Port-tariffs-2025, Port of Rotterdam, tariffs for third parties
f_{kp}	fixed cost of distribution center k for product p	2898.00	EUR	Port-tariffs-2025, Port of Rotterdam, tariffs for third parties
Capacities (for nominal scenario):				
g_{ip}^t	the supplier i capacity of product p for month t	It is calculated by taking the average historical proportion of product p produced in North or South America and multiplying it by the total demand for product p in all countries under consideration, taking into account the transformation process. This is then multiplied by the price of product p in that month and divided by the sum of the prices for all months for product p.	kton	FAOSTAT 2024; IndexMundi 2025

Table S2: Metadata for calculation of parameters part 2 of 3.

Parameters	Meaning	Value or Calculation	Unit	Sources
Capacities (for nominal scenario):				
e^t_{kp}	the distribution center (transhipment port) k capacity for product p for month t	It is calculated using the total demand for product p in all the countries under consideration. This figure is then multiplied by the price of product p in that month and divided by the sum of the prices for all months for product p.	kton	FAOSTAT 2024; IndexMundi 2025
Variable costs (for nominal scenario):				
$st_{jkl_{jk}}$	variable cost of transport combination mode l_{jk} associated with inflow and outflow operations from distribution center k to destination j	2310.00	EUR/kton	Prices table SILOPOR - 2025 (Portugal).
c_{ikp}	the intermodal transportation combination cost between supplier i and distribution center k for product p	0.001758206 divided by 1.852 (conversion of knots to km), multiplied by 1000 (conversion to kton), multiplied by the distance between i and k.	EUR/kton	Adapted from Bernacki (2021), using GDP deflator 2010-2024Q1 (Panamax).

Table S3: Metadata for calculation of parameters part 3 of 3.

Parameters	Meaning	Value or Calculation	Unit	Sources
Variable costs (for nominal scenario):				
$r_{jkl_{jk}}$	the unit cost of transportation mode combination l_{jk} between distribution center k and destination j	0.075 times the distance in km of roads, plus 0.040 times the distance in km of rails, plus 0.004036444 divided by 1.852, multiplied by 1000 times the distance in km of sea by handy-size.	EUR/kton	Adapted from Bernacki (2021), using GDP deflator 2010-2024Q1 (handy-size)
fc_k	the cost for using a distribution center k	6800.00	EUR/kton	Prices table SILOPOR - 2025 (Portugal)
Greenhouse gas emissions (for nominal scenario):				
ex_{ik}	the CO_2-equivalent emissions for transportation from origin i to distribution center k	0.01614 times the distance between i and k.	ton/kton	Climatiq 2025.
$ey_{jkl_{jk}}$	the CO_2-equivalent emissions for transportation from distribution center k to destination j using transport mode combination l_{jk}	0.0236 times the distance in km by roads, plus 0.017 times the distance in km by rails, plus 0.01614 times the distance by handy-size (sea).	ton/kton	Climatiq 2025.
